# Constitutive activation of WASp leads to abnormal cytotoxic cells with increased granzyme B and degranulation response to target cells

**DOI:** 10.1172/jci.insight.140273

**Published:** 2021-03-22

**Authors:** Joanna S. Kritikou, Mariana M.S. Oliveira, Julien Record, Mezida B. Saeed, Saket M. Nigam, Minghui He, Marton Keszei, Arnika K. Wagner, Hanna Brauner, Anton Sendel, Saikiran K. Sedimbi, Stamatina Rentouli, David P. Lane, Scott B. Snapper, Klas Kärre, Peter Vandenberghe, Jordan S. Orange, Lisa S. Westerberg

**Affiliations:** 1 Department of Microbiology Tumor and Cell Biology, Biomedicum C7, and; 2 Department of Medicine, Solna, Division of Rheumatology, Center for Molecular Medicine, Karolinska Institutet, Stockholm, Sweden.; 3 Gastroenterology Division, Children’s Hospital, Harvard Medical School, Boston, Massachusetts, USA.; 4 Center for Human Genetics, University Hospital Leuven, Leuven, Belgium.; 5 Department of Pediatrics, NewYork-Presbyterian Morgan Stanley Children’s Hospital, Columbia University Vagelos College of Physicians and Surgeons, New York, New York, USA.

**Keywords:** Immunology, Cellular immune response, Cytoskeleton, NK cells

## Abstract

X-linked neutropenia (XLN) is caused by gain-of-function mutations in the actin regulator Wiskott-Aldrich Syndrome protein (WASp). XLN patients have reduced numbers of cytotoxic cells in peripheral blood; however, their capacity to kill tumor cells remains to be determined. Here, we examined NK and T cells from 2 patients with XLN harboring the activating WASp^L270P^ mutation. XLN patient NK and T cells had increased granzyme B content and elevated degranulation and IFN-γ production when compared with healthy control cells. Murine WASp^L272P^ NK and T cells formed stable synapses with YAC-1 tumor cells and anti-CD3/CD28–coated beads, respectively. WASp^L272P^ mouse T cells had normal degranulation and cytokine response whereas WASp^L272P^ NK cells showed an enhanced response. Imaging experiments revealed that while WASp^L272P^ CD8^+^ T cells had increased accumulation of actin upon TCR activation, WASp^L272P^ NK cells had normal actin accumulation at lytic synapses triggered through NKp46 signaling but had impaired response to lymphocyte function associated antigen-1 engagement. When compared with WT mice, WASp^L272P^ mice showed reduced growth of B16 melanoma and increased capacity to reject MHC class I–deficient cells. Together, our data suggest that cytotoxic cells with constitutively active WASp have an increased capacity to respond to and kill tumor cells.

## Graphical abstract

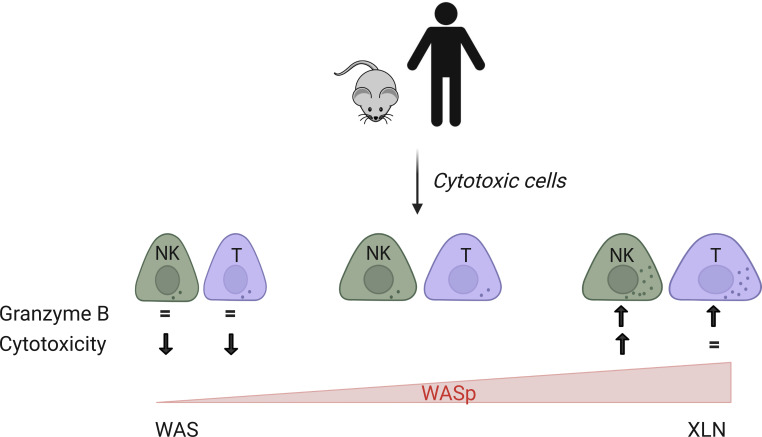

## Introduction

Patients with X-linked neutropenia (XLN) have severe congenital neutropenia, recurrent infections, and a higher susceptibility to developing hematological malignancies (1, 2). XLN patients have a low number of natural killer (NK) cells and a reversed ratio of CD4^+^ and CD8^+^ T cells in peripheral blood (1, 2). The disease is caused by gain-of-function mutations in the actin regulator Wiskott-Aldrich Syndrome protein (WASp). WASp is uniquely expressed in hematopoietic cells and critically dependent on its structural conformation for activity (3–6). At rest, WASp resides in an autoinhibited folding due to interaction of the GTPase binding domain (GBD) and the verprolin-cofilin-acidic (VCA) domain. Upon interaction with the small GTPase cell division cycle 42 (Cdc42) to the GBD, the autoinhibition is released to expose the VCA domain that interacts with the actin related protein 2/3 (Arp2/3) complex to induce actin polymerization leading to a branched network of actin filaments (3–7). Four XLN mutations in WASp have been identified to date, L270P, S272P, I290T, and I294T (1, 2, 8, 9). All XLN mutations are localized in the GBD and induce structural changes in WASp leading to a constitutively active WASp in vitro and in vivo (1, 2, 8, 10–12). Moreover, as predicted from biochemical studies of WASp and the close homolog neuronal WASp (N-WASp) (5), XLN mutations induce increased phosphorylation of a critical tyrosine-291 (murine tyrosine-293), even in the absence of receptor activation (11). Phosphorylated WASp has prolonged activity, also after dissociation from Cdc42, and can be activated by Src family kinases and Tec family kinases (5, 13, 14). Neutrophils and B and T cells expressing XLN mutations in WASp show increased F-actin content and decreased capacity to form firm adhesion upon receptor activation (2, 10–12, 15). XLN mutations lead to genomic instability of B cells and myeloid cells, likely caused by accumulation of F-actin during cytokinesis, increased mechanical stress at the kinetochore, and activation of Aurora B kinase error correction (10, 12, 16). The role of cytotoxic cells in XLN disease pathogenesis and immune tumor surveillance remains largely unknown. To examine if XLN mutations induced increased tumor susceptibility, we previously bred WASp^–/–^ mice and XLN murine models (WASp^L272P^ and WASp^I296T^) to p53 heterozygous background (15). While mice devoid of WASp expression had earlier tumor onset and mortality, we found surprisingly in the context of genomic instability of XLN B cells that XLN mice show delayed tumor onset when compared with WT mice (15).

NK cell responses are regulated by germline-encoded activating and inhibitory receptors that sense activating ligands on the target cells and/or loss of major histocompatibility complex (MHC) class I (17–19). The net input of signaling from the activating and inhibitory receptors determines when the NK cell elicits a response to the target cell, such as tumor cells and virally infected cells, and when the NK cell remains tolerant to healthy cells that express inhibitory ligands and MHC class I molecules (17–19). In contrast to NK cells that recognize loss of MHC class I molecules, cytotoxic CD8^+^ T cells (CTLs) form an immune synapse based on T cell receptor (TCR) recognition of MHC class I molecule–peptide on the target cells. NK cells and CTLs form a lytic immune synapse consisting of polymerized actin, the microtubule organizing center polarized toward the synapse, and lytic granules containing pore-forming perforin and lytic granzymes that are released into the synaptic cleft to kill the target cell (20–23). The peripheral synapse consists of a densely packed actin network containing branched actin fibers and discrete actin foci whereas the central synapse has a less dense actin network where filament displacement leads to stochastic clearance formation and disappearance that allows release of lytic granules in the pores of the actin network (24–26). Actin dynamics is mediated by Arp2/3 regulated by WASp and the WASp family member WAVE2, and contractility forces are generated by actin-myosin IIA (24, 25). NK cells can use as little as 2 to 4 degranulation events to mediate target cell death (27).

WASp serves an important role for functionality of cytotoxic cells as evident in patients devoid of WASp with the severe immunodeficiency Wiskott-Aldrich syndrome (WAS, ref. 28) and in mouse models of WAS where WASp is deleted by gene targeting (29). NK cells devoid of WASp show decreased capacity to form the lytic immune synapse and have reduced capacity to kill tumor cells in vitro and in vivo (15, 30–34). Interestingly, IL-2 treatment of WASp-deficient NK cells rescues the defective killing capacity, and IL-2 naturally produced by tumor cells increases the killing capacity by WASp-deficient NK cells (31, 33–35). This has prompted clinical trials for testing if IL-2 treatment can rescue aberrant NK cell functionality in nontransplanted WAS patients, which have obtained promising results (35). Moreover, WASp deficiency leads to decreased NK cell migration in response to the activating receptor natural killer group 2D (NKG2D) (36), and this is likely to influence the recruitment of WASp-deficient NK cells to the site of infection or tumor mass. CTLs devoid of WASp fail to organize the peripheral actin cluster of the cytolytic synapse rich in the adhesion molecule lymphocyte function associated antigen-1 (LFA-1), and many of the WASp-deficient CTLs show breakage of the dense actin ring symmetry, resulting in delayed target cell killing in vitro and in vivo upon viral infection (37–39).

We hypothesized that NK cells and CTLs with constitutively active WASp in XLN have altered capacity to remodel the actin cytoskeleton, affecting their capacity to kill tumor cells. We examined cells from 2 patients with XLN expressing the WASp^L270P^ mutation and a mouse model that we recently generated that expresses the corresponding murine XLN mutation WASp^L272P^. XLN patient NK cells and CTLs had markedly elevated expression of granzyme B and increased degranulation and IFN-γ production, suggesting that XLN patient NK cells and CTLs cells had an effector phenotype. Murine WASp^L272P^ NK cells and CTLs showed increased degranulation and IFN-γ production upon engagement of activating receptors, as well as increased polarization of polymerized actin toward the substratum in the absence of receptor stimulation. When compared with WT mice, WASp^L272P^ mice showed increased infiltration of NK and T cells in the tumor, and this was associated with delayed B16 melanoma tumor growth. Our data show that NK cells and CTLs in XLN had a hyperactive phenotype, suggesting that increased WASp activity and actin polymerization may be beneficial in cytotoxic cells for eradication of tumor cells.

## Results

### XLN patients have altered NK and T cell populations that display increased granzyme B content.

**Figure 1:**
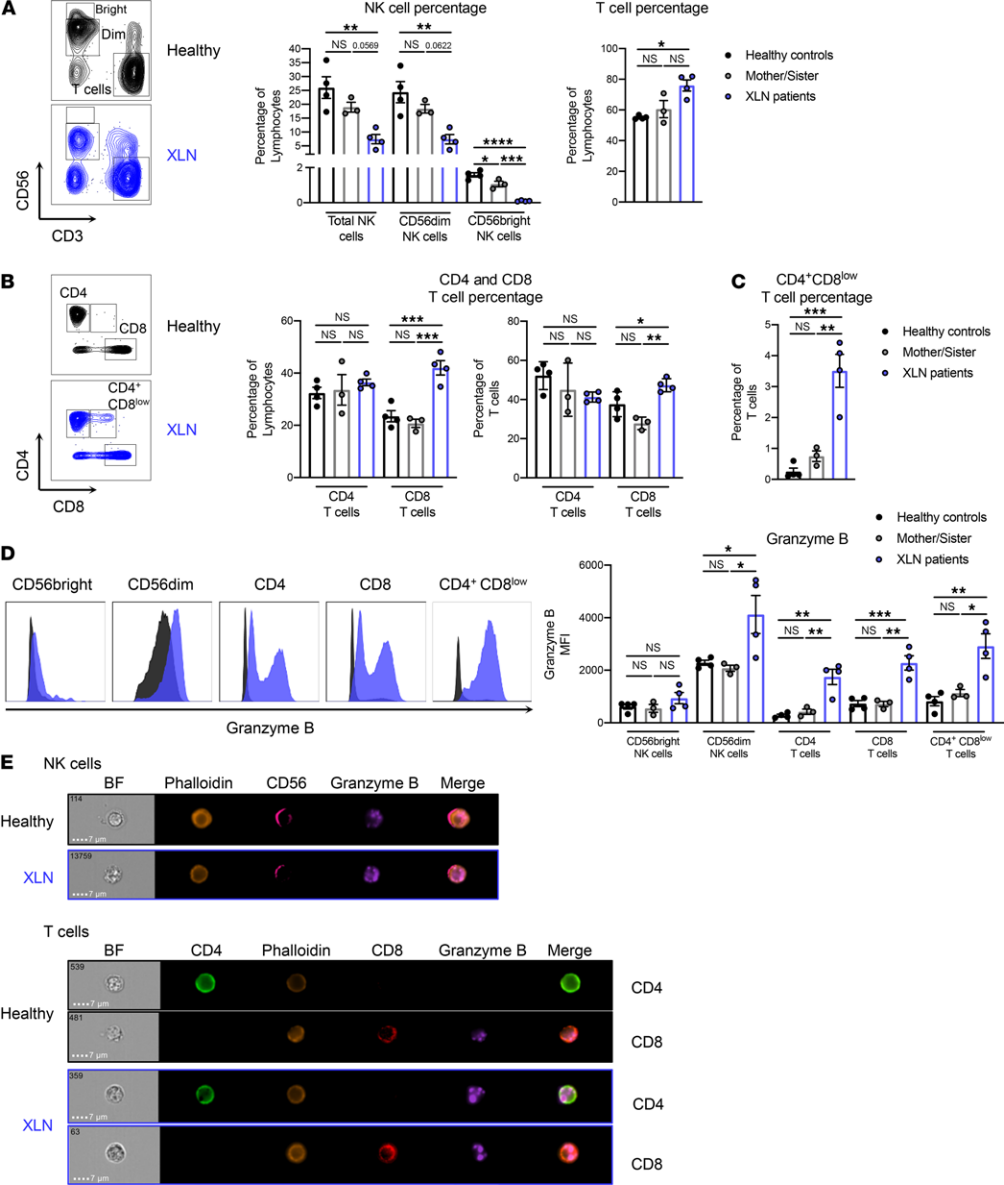
XLN patients have altered NK and T cell populations that display increased granzyme B content. (**A**) CD56 and CD3 and (**B**) CD4 and CD8 staining on PBMCs from 2 XLN patients, the mother and the sister of the patients, and 2 healthy controls. Representative flow cytometry plots are shown at left and graphs of NK and T cells at right. (**C**) The percentage of the CD3^+^CD4^+^CD8^lo^ T cell population in the 2 XLN patients, the mother and the sister of the patients, and 2 healthy controls. (**D**) Granzyme B expression in CD56^bright^ and CD56^dim^ NK cells and CD4^+^, CD8^+^, and CD3^+^CD4^+^CD8^lo^ T cells. Representative flow cytometry plots are shown at left and graphs of the granzyme content in NK and T cells of XLN patients and controls at right. (**E**) Imaging flow cytometry of the granzyme B granules in NK cells (*top*) and CD4^+^ and CD8^+^ T cells (*bottom*) from the XLN patients and healthy controls. Representative images of bright-field (BF) and the fluorescent markers are shown. The data represent 2 individual experiments combined. Graphs show mean values ± SEM and significance was assessed by 1-way ANOVA. *P ≤ 0.05, **P ≤ 0.01, ***P ≤ 0.001.

NK cytopenia has previously been observed in members of the family with XLN WASp^L270P^ studied here, where NK cell numbers ranged between 11 × 10^6^/L and 110 × 10^6^/L (*n* = 3 patients) in peripheral blood (1). The normal range for blood NK cells is 150 × 10^6^/L to 400 × 10^6^/L. In another study of a large family harboring the XLN WASp^I294T^ mutation, the NK cell number ranged between 63 × 10^6^/L and 173 × 10^6^/L (*n* = 10 patients) in peripheral blood (8). Together, these observations suggest that overactive WASp mutations in XLN lead to NK cytopenia. To examine the phenotype and functionality of cytotoxic lymphocytes in XLN, we collected blood from 2 brothers with the WASp^L270P^ mutation (XLN patients), their mother and sister (mother/sister), and 2 healthy donors. PBMCs were isolated, and NK cells and CD4^+^ and CD8^+^ T cells were analyzed by flow cytometry (the gating strategy is shown in Supplemental Figure 1). When compared with their mother, their sister, and the healthy controls, the patients with XLN had a lower percentage of total NK cells and CD56^dim^ NK cells while the CD56^bright^ NK cell population was absent (Figure 1A). The percentage of T cells was higher in the patients with XLN when compared with healthy controls (Figure 1A). As previously shown (1), the proportion of CD8^+^ T cells was increased in the patients with XLN, leading to a skewed CD4^+^/CD8^+^ T cell ratio when compared with the healthy controls (Figure 1B). Interestingly, the XLN patient T cells contained a population of CD3^+^CD4^+^CD8^lo^ cells, not detected in any of the other groups (Figure 1C). XLN patient NK and T cells also had altered receptor expression. CD4^+^ and CD8^+^ T cells had increased expression of inhibitory killer cell lectin-like receptor G1 (KLRG1; Supplemental Figure 2A; supplemental material available online with this article; https://doi.org/10.1172/jci.insight.140273DS1), while NK cells showed lower KLRG1 expression (Supplemental Figure 2B), when compared with healthy controls. CD69 and DNAX accessory molecule-1 (DNAM-1) expression was unaltered between XLN patients and healthy controls in all cell types examined (Supplemental Figure 2, A and B). To assess the effector potential of the XLN patient lymphocytes, we examined the expression of the effector molecule granzyme B by flow cytometry and by imaging flow cytometry in NK and T cells. CD56^dim^ NK cells, CD4^+^ and CD8^+^ T cells, as well as the CD4^+^CD8^lo^ T cell population from the patients with XLN had significantly elevated expression of granzyme B (Figure 1, D and E). In contrast, only low granzyme B was detected in NK cells and T cells of healthy controls (Figure 1, D and E).

**Figure 2:**
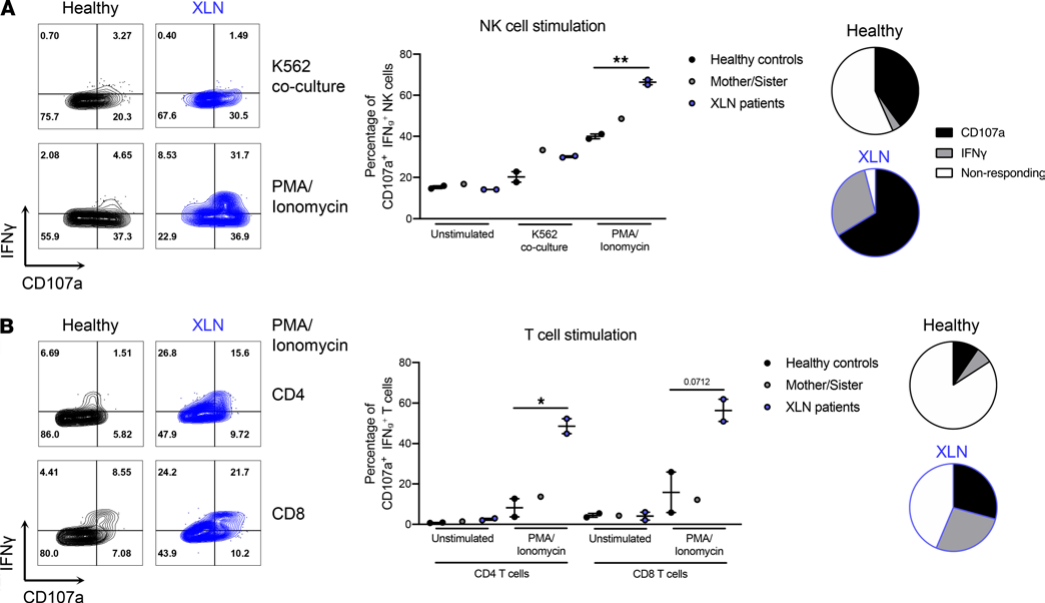
NK and T cells from XLN patients are more responsive to stimulation. (**A**) Degranulation, as measured by CD107a surface expression, and IFN-γ production in healthy control and XLN patient NK cells. Representative flow cytometry plots are shown on the left and quantification of NK cell responses against K562 tumor cells and PMA/Ionomycin on the right. CD107a^+^IFN-γ^+^ NK cells include CD107a single positive (SP), IFN-γ SP, and CD107a IFN-γ double-positive (DP) cells. The average of nonresponding and CD107a/IFN-γ–responding NK cells after PMA/ionomycin stimulation is shown as pie charts in healthy controls and XLN patients. (**B**) Degranulation and IFN-γ production in healthy control and XLN patient CD4^+^ and CD8^+^ T cells. Representative flow cytometry plots are shown on the left and quantification of T cell responses to PMA/ionomycin stimulation on the right. CD107a^+^IFN-γ^+^ T cells include CD107a SP, IFN-γ SP, and CD107a IFN-γ DP cells. The average of nonresponding and CD107a/IFN-γ–responding CD8^+^ T cells after PMA/ionomycin stimulation is shown as pie charts in healthy controls and XLN patients. One representative experiment of 2 is shown, with 2 samples for the healthy and XLN groups and 1 sample for the mother/sister control group. Graphs show mean values ± SEM and significance was assessed by 2-tailed Student’s *t* test between the healthy control and XLN patient groups. *P ≤ 0.05, **P ≤ 0.01. If no other indication, results were not significant.

To assess their capacity to respond to stimulation, NK cells from the patients with XLN, their mother and sister, and healthy controls were cocultured with K562 myelogenous leukemia cells, after which degranulation and cytokine production were measured by flow cytometry. When compared with healthy controls, XLN patient NK cells had a slight increase in degranulation against K562 cells, as demonstrated by increased CD107a surface expression (Figure 2A). When stimulated with PMA/ionomycin, which bypasses specific receptor activation, the XLN patient NK cells showed a significantly increased IFN-γ and CD107a response, when compared with healthy control NK cells (Figure 2A). We next assessed the response of XLN patient T cells to PMA and ionomycin. XLN CD4^+^ and CD8^+^ T cells also showed increased CD107a degranulation and IFN-γ production, when compared with healthy control T cells (Figure 2B).

Together, these results suggest that XLN patient NK and T cells have an altered subset distribution in peripheral blood and that these cells display signs of hyperactivity, with increased intracellular quantity of the effector molecules granzyme B and IFN-γ and elevated degranulation capacity.

### Murine WASp^L272P^ NK cells mature and are educated normally but display increased degranulation and IFN-γ production.

To examine the XLN NK cell and T cell responses in peripheral organs, we used a mouse model expressing the WASp^L272P^ mutation (11). The murine WASp^L272P^ mutation corresponds to the human WASp^L270P^ mutation and is part of the WASp GBD with 100% amino acid identity in mice and humans (11). WT and WASp^L272P^ mice showed a similar number of NK cells in the spleen (Supplemental Figure 3A) and no difference in total F-actin content when compared with WT cells (Supplemental Figure 3B). By using markers CD27 and CD11b, differentiation stages of NK cells in the spleen can be examined (40, 41). WT and WASp^L272P^ mice had similar composition of the CD27^–^CD11b^–^, CD27^+^CD11b^–^, CD27^+^CD11b^+^, and CD27^–^CD11b^+^ NK cell populations in the spleen (Supplemental Figure 4A). Moreover, WT and WASp^L272P^ NK cells had similar expression of activating/costimulatory receptors Ly49D, Ly49H, NK1.1, NKp46, NKG2D, DNAM-1, 2B4, DX5, LFA-1, CD69, CD25, and CD122 (Supplemental Figure 4A) and of inhibitory receptors Ly49A, Ly49C, Ly49G2, Ly49I, NKG2A, CTLA-4, programmed cell death protein-1 (PD-1), and LAG3 (Supplemental Figure 4B). When compared with WT NK cells, WASp^L272P^ NK cells showed lower expression of the inhibitory receptor KLRG1 (Supplemental Figure 4B), similar to what we observed in the XLN patient NK cells (Supplemental Figure 2B). We next examined whether WASp^L272P^ NK cells were normally educated by assessing the frequency of NK cells expressing inhibitory receptors specific to self–MHC class I Ly49C (C), Ly49I (I), or NKG2A (N) or any combination of the 3 (C+I+N+CI+CN+IN+CIN) versus NK cells expressing non–self-specific inhibitory receptors Ly49A (A) or Ly49G2 (G) or any combination thereof (A+G+AG, refs. 33, 42). A high proportion of NK cells (~50%) from WT and WASp^L272P^ mice expressed inhibitory receptors Ly49C, Ly49I, and NKG2A that recognize self H-2^b^ MHC class I molecules in C57BL/6 mice (CIN, Supplemental Figure 4C). Less than 10% of NK cells from WT and WASp^L272P^ mice expressed any of the non–self-specific inhibitory receptors Ly49A and Ly49G2 that lack MHC class I ligand in C57BL/6 hosts (AG, Supplemental Figure 4C). One of the key features of an educated NK cell receptor repertoire in MHC class I–sufficient mice is a shift toward a repertoire where many NK cells express only 1–2 inhibitory receptors (42). WT and WASp^L272P^ NK cells had similar frequency of cells expressing 1–2 inhibitory receptors (Supplemental Figure 4D). These data suggest that WASp^L272P^ NK cells were educated correctly in vivo with regard to inhibitory receptor expression.

**Figure 3:**
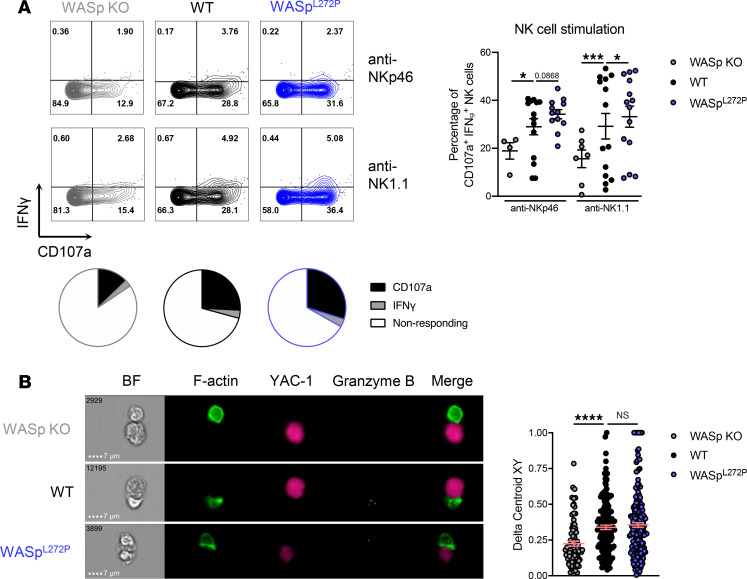
Murine WASp^L272P^ NK cells have increased degranulation and IFN-γ production and normal synapse formation. (**A**) Degranulation and IFN-γ production in NK cells from WASp-KO, WT, and WASp^L272P^ mice upon cross-linking of the activating receptors NKp46 or NK1.1 with antibodies. Representative plots of the IFN-γ production and degranulation (CD107a) in NK cells with anti-NKp46 (*top*) and anti-NK1.1 (*bottom*) stimulation are shown on the left and a cumulative graph of 4 experiments on the right. CD107a^+^IFN-γ^+^ NK cells include CD107a single positive (SP), IFN-γ SP, and CD107a IFN-γ double-positive (DP) cells. Each dot represents 1 mouse. WASp-KO anti-NKp46 *n* = 4, WT anti-NKp46 *n* = 14, WASp^L272P^ anti-NKp46 *n* = 12, WASp-KO anti-NK1.1 *n* = 7, WT anti-NK1.1 *n* = 14, WASp^L272P^ anti-NK1.1 *n* = 14. Graph shows mean values ± SEM and significance was assessed by 2-tailed Student’s *t* test. The average of nonresponding and CD107a/IFN-γ–responding NK cells after NK1.1 stimulation is also shown as pie charts in each genotype. (**B**) Imaging flow cytometry to assess synapse formation between NK cells from WASp-KO, WT, or WASp^L272P^ mice and SNARF-1–stained YAC-1 lymphoma cells (pink). Doublets were selected as a function of area and aspect ratio and conjugates as a function of NK1.1 and SNARF-1 intensity. All conjugates were confirmed to consist of 1 NK cell and 1 YAC-1 lymphoma cell. Actin is shown in green (LifeActGFP) and granzyme B in orange. Representative images of conjugates are shown on the left, and a representative experiment of 3 is shown on the right. The Delta Centroid value indicates the extent of polarized fluorescence toward the synapse. Each dot represents 1 conjugate. WASp-KO *n* = 82, WT *n* = 161, WASp^L272P^ *n* = 203. Graph shows mean values ± SEM and significance was assessed by 1-way ANOVA. *P ≤ 0.05, ***P ≤ 0.001, ****P ≤ 0.0001.

We next tested the capacity of WASp^L272P^ NK cells to degranulate and produce IFN-γ upon stimulation via activating receptors NKp46 and NK1.1. WASp-KO NK cells showed a reduced response, as previously shown (33), whereas WASp^L272P^ NK cells showed increased degranulation and IFN-γ production, when compared with WT NK cells (Figure 3A). Baseline degranulation and IFN-γ production were similar between WT and WASp^L272P^ NK cells, as was their response to PMA/ionomycin stimulation (Supplemental Figure 3C). Additionally, naive WT and WASp^L272P^ NK cells had a similar granzyme B and perforin content (Supplemental Figure 4G). To test the effectiveness of these degranulation events, we incubated NK cells with YAC-1 lymphoma cells and monitored target cell death by live cell imaging and in vivo YAC-1 rejection during 48 hours. WT and WASp^L272P^ NK cells showed similar killing of YAC-1 lymphoma cells in vitro and in vivo (Supplemental Figure 5, A and B, respectively). Functional lytic synapses between NK cells and tumor cells are characterized by polarization of F-actin toward the synapse interface, as well as localization of granzyme B^+^ lytic granules at the synapse. To examine NK cell synapse formation, WT, WASp-KO, and WASp^L272P^ NK cells were cocultured with YAC-1 lymphoma cells, and thereafter conjugate formation was analyzed by ImageStream imaging flow cytometry (Figure 3B). NK cells devoid of WASp had a reduced capacity to polarize F-actin toward the immunological synapse, as previously reported (refs. 30, 31, 33, 34 and Figure 3B). WASp^L272P^ NK cells polarized F-actin toward the YAC-1 lymphoma cells similarly to WT NK cells, as measured by the Delta Centroid analysis (Figure 3B).

These data suggest that not only were WASp^L272P^ NK cells functional, in fact, they also showed signs of hyperactivity with increased responses to stimulation.

### T cells from WASp^L272P^ mice respond to stimulation and form lytic synapses.

**Figure 4:**
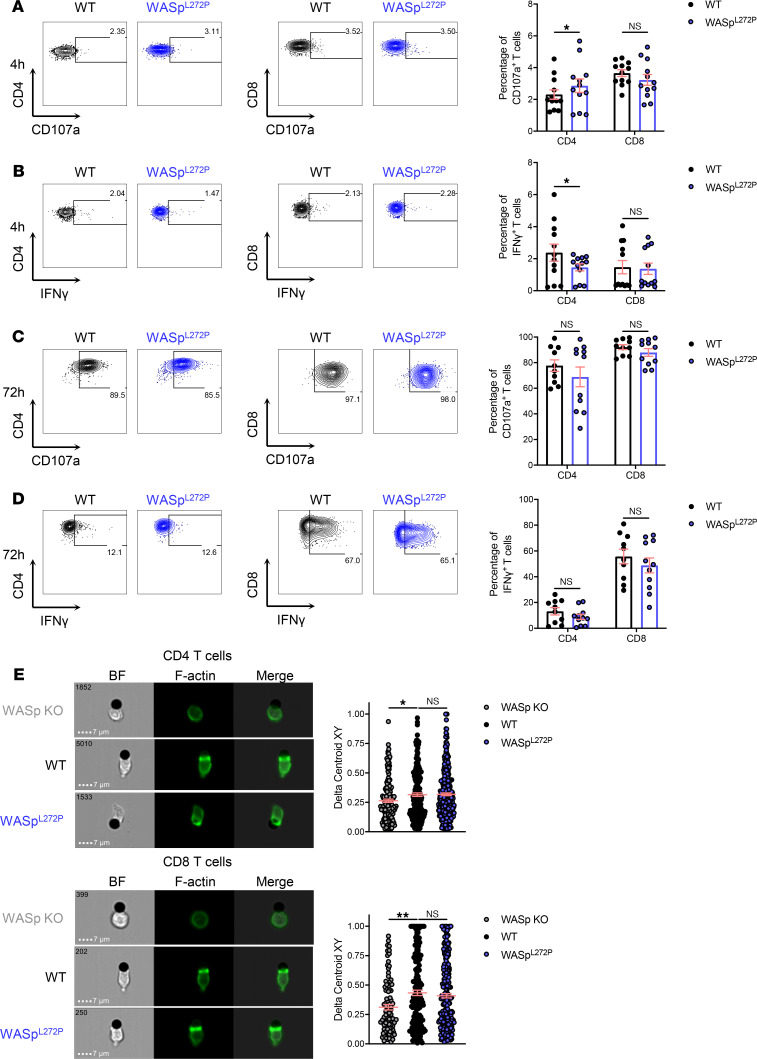
T cells from WASp^L272P^ mice respond to stimulation and form lytic synapses. (**A**) Degranulation in CD4^+^ and CD8^+^ T cells from WT and WASp^L272P^ mice after a 4-hour anti-CD3 and anti-CD28 stimulation. (**B**) IFN-γ production in CD4^+^ and CD8^+^ T cells from WT and WASp^L272P^ mice after a 4-hour anti-CD3 and anti-CD28 stimulation. Representative plots are shown on the left, and quantification of 4 experiments is shown on the right. Each dot represents 1 mouse. WT *n* = 12, WASp^L272P^ *n* = 12. (**C**) Degranulation in CD4^+^ and CD8^+^ T cells from WT and WASp^L272P^ mice after a 72-hour anti-CD3 and anti-CD28 stimulation. (**D**) IFN-γ production in CD4^+^ and CD8^+^ T cells from WT and WASp^L272P^ mice after a 72-hour anti-CD3 and anti-CD28 stimulation. Representative plots are shown on the left, and quantification of 4 experiments is shown on the right. Each dot represents 1 mouse. WT *n* = 10, WASp^L272P^ *n* = 11. Graphs show mean values ± SEM and significance was assessed by 2-tailed Student’s *t* test and the Mann-Whitney correction. (**E**) Synapse formation between WASp-KO, WT, and WASp^L272P^ CD4^+^ T cells (*top*) and CD8^+^ T cells (*bottom*) with anti-CD3/CD28–coated beads. Actin is shown in green (LifeActGFP). The Delta Centroid value indicates the extent of polarized fluorescence towards the synapse. A representative experiment of 3 is shown. Each dot represents 1 conjugate. CD4^+^ WASp-KO *n* = 165, WT *n* = 200, WASp^L272P^ *n* = 303, CD8^+^ WASp-KO *n* = 100, WT *n* = 185, WASp^L272P^ *n* = 258. Graphs show mean values ± SEM and significance was assessed by 1-way ANOVA. *P ≤ 0.05.

We next investigated the responsiveness of CD4^+^ and CD8^+^ T cells in the XLN mouse model. WT and WASp^L272P^ CD4^+^ and CD8^+^ T cells had similar expression of activating/costimulatory receptors LFA-1 and CD69 (Supplemental Figure 4, E and F) and of inhibitory receptors CTLA-4, PD-1, LAG3, and KLRG1 (Supplemental Figure 4, E and F). WT and WASp^L272P^ mice had similar composition of naive CD44^lo^CD62L^hi^, effector CD44^lo^CD62L^lo^, and memory CD44^hi^CD62L^lo^ CD4^+^ and CD8^+^ T cells in the spleen (Supplemental Figure 4, E and F) and similar expression of granzyme B and perforin in CD4^+^ and CD8^+^ T cells (Supplemental Figure 4G). WT and WASp^L272P^ T cells showed similar degranulation and IFN-γ production when stimulated with plate-bound anti-CD3 and anti-CD28 antibodies for 4 hours and 72 hours (Figure 4, A–D), as well as at baseline and after PMA/ionomycin stimulation (Supplemental Figure 3, D and E, respectively). To elucidate whether T cells from the XLN mouse model could accumulate F-actin at the synapse, we incubated them with beads coated with anti-CD3 and anti-CD28 antibodies. CD4^+^ and CD8^+^ T cells from WT and WASp^L272P^ mice polarized F-actin toward the activating beads (Figure 4E), whereas WASp-KO T cells had reduced capacity to polarize F-actin (Figure 4E and ref. 33). We next examined the T cell capacity to kill A20 lymphoma cells, coated with anti-CD3 and anti-CD28 antibodies, by live cell imaging. When compared with WT, WASp^L272P^ CD4^+^ and CD8^+^ T cells had a similar capacity to kill A20 lymphoma cells via TCR stimulation (Supplemental Figure 5C).

These data suggest that WASp^L272P^ mice had normal CD4^+^ and CD8^+^ T cell responses in vitro.

### Dysregulated F-actin polymerization at WASp^L272P^ XLN NK and CD8^+^ T cell synapses.

**Figure 5:**
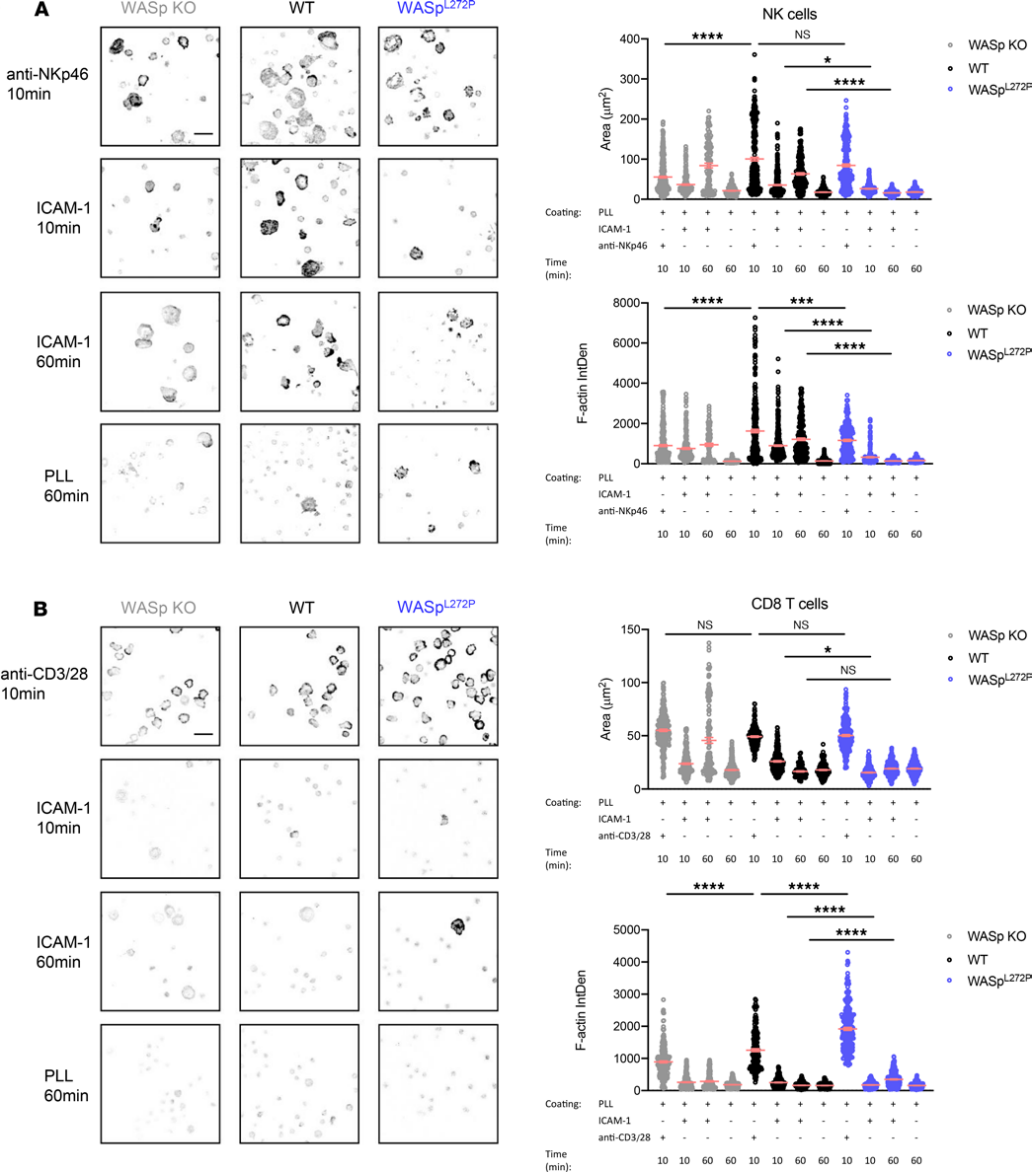
NK and CD8^+^ T cells from WASp^L272P^ mice have a different spreading and F-actin polarizing response on coated glass. (**A** and **B**) Confocal images of WASp-KO, WT, and WASp^L272P^ (**A**) NK cells and (**B**) CD8^+^ T cells to assess attachment and F-actin density toward the indicated coated surface. Representative images are shown (*left*) and graphs quantifying the attachment area (*top right*) and F-actin density by confocal imaging (*bottom right*) in arbitrary units. Images are displayed as inverted gray scale Lookup Table of phalloidin staining. Two individual experiments combined are shown. Each dot represents 1 cell. For each condition, between 200 and 400 cells were analyzed. Graphs show mean values ± SEM and significance was assessed by 1-way ANOVA. *P ≤ 0.05, ***P ≤ 0.001, ****P ≤ 0.0001.

To interrogate possible WASp-dependent differences in actin rearrangements during lytic synapse formation between NK cells and CD8^+^ T cells, we used ligand-coated surfaces and quantitative confocal microscopy to measure actin accumulation in response to activating receptors or recombinant ligand for the adhesion receptor LFA-1. LFA-1 interaction with ICAM-1 modulates lytic synapse formation by both NK and T cells (43, 44). NK cells were incubated on glass coverslips coated with anti-NKp46 antibody for 10 minutes, recombinant mouse ICAM-1 (to engage LFA-1) for 10 and 60 minutes, or poly-l-lysine for 60 minutes. WT NK cells spread and accumulated F-actin at the interphase in response to anti-NKp46 but not to ICAM-1 by 10 minutes (Figure 5A). This was not due to differences in frequency of responding cells because a similar number of cells adhered to these surfaces (Supplemental Figure 6). As previously shown, WASp-KO NK cells had a reduced spreading and actin accumulation in response to NKp46 engagement (Figure 5A) (33). Upon NKp46 activation, WASp^L272P^ NK cells spread and accumulated F-actin to WT NK cell levels (Figure 5A). Interestingly, both WT and WASp-KO NK cells adhered to ICAM-1–coated surfaces and had increased spreading and actin accumulation, which was pronounced by 60 minutes. However, WASp^L272P^ NK cells failed to respond to this stimulation (Figure 5A and Supplemental Figure 6). Since the degranulation response was examined after 4 hours (Figure 3A), we assessed the long-term response of WASp^L272P^ NK cell stimulation. Both WT and WASp^L272P^ NK cells adhered and accumulated F-actin toward the interface in response to NKp46 stimulation at 4 hours (Supplemental Figure 7, A and B), as they did after 10 minutes. However, while WT NK cells did not respond in the absence of anti-NKp46, WASp^L272P^ NK cells showed an increased accumulation of F-actin and adhesion toward the surface devoid of anti-NKp46 (Supplemental Figure 7, A and B). Together with the reduced response to LFA-1 engagement, this suggests that WASp^L272P^ NK cells have dysregulated F-actin dynamics. To examine CD8^+^ T cell responses, CD8^+^ T cells were stimulated on anti-CD3+anti-CD28 antibody–coated coverslips for 10 minutes or with recombinant mouse ICAM-1 for 10 and 60 minutes. When compared with WT CD8^+^ T cells, WASp-KO CD8^+^ T cells spread less and accumulated less actin (Figure 5B). WASp^L272P^ CD8^+^ T cells on the other hand showed enhanced spreading and actin accumulation when compared with WT cells (Figure 5B). WT CD8^+^ T cells spread on and accumulated F-actin poorly to nonactivating surfaces in general. However, they showed a brief response to short-term LFA-1 stimulation. This response was delayed in WASp^L272P^ T cells and greatly enhanced by 60 minutes in WASp-KO T cells (Figure 5B). These data indicate altered actin dynamics in response to TCR/CD28 or LFA-1 engagement by WASp^L272P^ CD8^+^ T cells. Taken together, these data suggest that WASp deficiency reduces actin responses at lytic synapses formed by NK cells and CD8^+^ T cells while constitutive activation of WASp enhances actin accumulation at CD8^+^ T cell but not NK cell synapses. Moreover, constitutive activation of WASp leads to impaired responses to LFA-1 that are greatly pronounced in NK cells.

### WASp^L272P^ cells have a reconstitution advantage in vivo, and WASp^L272P^ mice have increased responsiveness against MHC class I–deficient hematopoietic cells.

**Figure 6:**
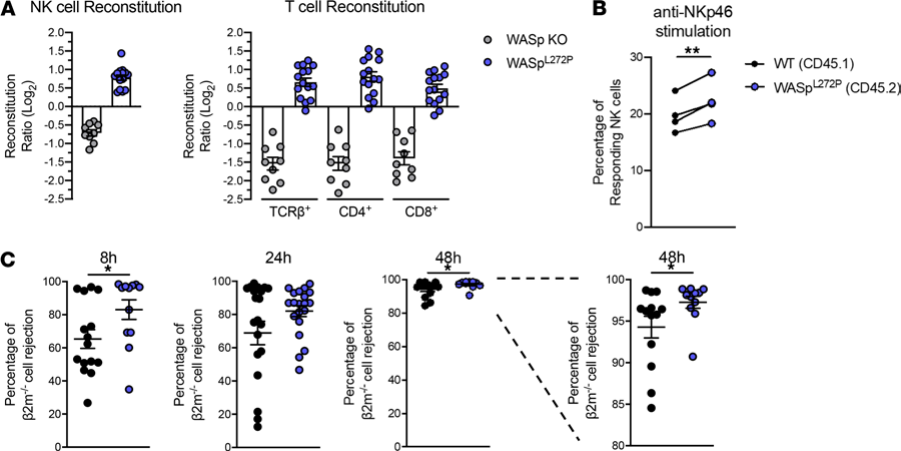
WASp^L272P^ NK and T cells have a reconstitution advantage in vivo, and WASp^L272P^ mice exhibit increased responsiveness against MHC class I–deficient hematopoietic grafts. (**A**) Ratios of CD45.2 WASp^L272P^/CD45.1 WT and CD45.2 WASp-KO/CD45.1 WT NK cells (*left*) and T cells (*right*) in spleens of mixed bone marrow chimera mice. Ratios were normalized by their original bone marrow ratio (CD45.2/CD45.1) and plotted on a logarithmic scale. The data represent 3 individual experiments combined. Each dot represents 1 mouse. WASp-KO/WT *n* = 9, WASp^L272P^/WT *n* = 15. Graph shows mean values ± SEM. (**B**) Stimulation of splenic NK cells from mice reconstituted with a mix of WT and WASp^L272P^ bone marrow cells. One representative experiment is shown, *n* = 4. Each dot represents 1 mouse and the lines connect NK cells from the same mouse. Graph shows mean values ± SEM and significance was assessed by paired 2-tailed Student’s *t* test. (**C**) Rejection of β2m^–/–^ splenocytes in WT and WASp^L272P^ mice after 8, 24, and 48 hours. Each dot represents 1 mouse. The data represent 3 individual experiments combined. 8 hours and 48 hours: WT *n* = 15, WASp^L272P^ *n* = 12; 24 hours: WT *n =* 21, WASp^L272P^ *n* = 20. Graphs show mean values ± SEM and significance was assessed by 2-tailed Student’s *t* test and the Mann-Whitney correction. *P ≤ 0.05, **P ≤ 0.01. If no other indication, results were not significant.

WASp deficiency has been termed a cell trafficking disorder because many hematopoietic cells show decreased migration to chemokines in vitro and homing to peripheral organs in vivo (28). In contrast, XLN mutations induce increased actin dynamics and migratory capacity of neutrophils, both in vitro and in vivo (11). To examine if WASp^L272P^ NK and T cells had a competitive advantage over WT cells, we established bone marrow chimeric mice where WT and WASp^L272P^ bone marrow cells were injected into lethally irradiated host mice. The congenic markers CD45.1 and CD45.2 were used to define WT and WASp^L272P^ or WASp-KO cells, respectively. WASp^L272P^ NK and T cells had a 2-fold advantage over WT cells to reconstitute the spleen (Figure 6A). In contrast, WASp-KO NK and T cells had a 2-fold disadvantage, when compared with WT cells (Figure 6A). When splenocytes from reconstituted mice were analyzed for degranulation and IFN-γ production in response to NKp46 stimulation side by side, WASp^L272P^ NK cells had a higher responsiveness, as compared with WT NK cells (Figure 6B and Supplemental Figure 5D). These data suggest an intrinsic effect of the WASp^L272P^ mutation on NK and T cells. To specifically address the in vivo functionality of NK cells in WASp^L272P^ mice, we performed a competitive assay in which we injected normal splenocytes (expressing MHC class I) and splenocytes that lack expression of MHC class I (β2m^–/–^), labeled with different concentrations of CFSE (33). Both WT and WASp^L272P^ mice could efficiently reject β2m^–/–^ splenocytes after 24 hours (Figure 6C). Interestingly, the rejection capacity of WASp^L272P^ mice was higher than that of WT mice at the earliest time point examined, with more than 80% of the mice tested having completely rejected the β2m^–/–^ cells after 8 hours, as compared with approximately 60% of the WT mice (Figure 6C). When compared with WT NK cells, WASp^L272P^ NK cells also had higher rejecting capacity of β2m^–/–^ cells at 48 hours (Figure 6C). This suggests that WASp^L272P^ NK cells had an increased “missing-self” rejection capacity.

### WASp^L272P^ mice show increased responsiveness against tumors in vivo.

**Figure 7:**
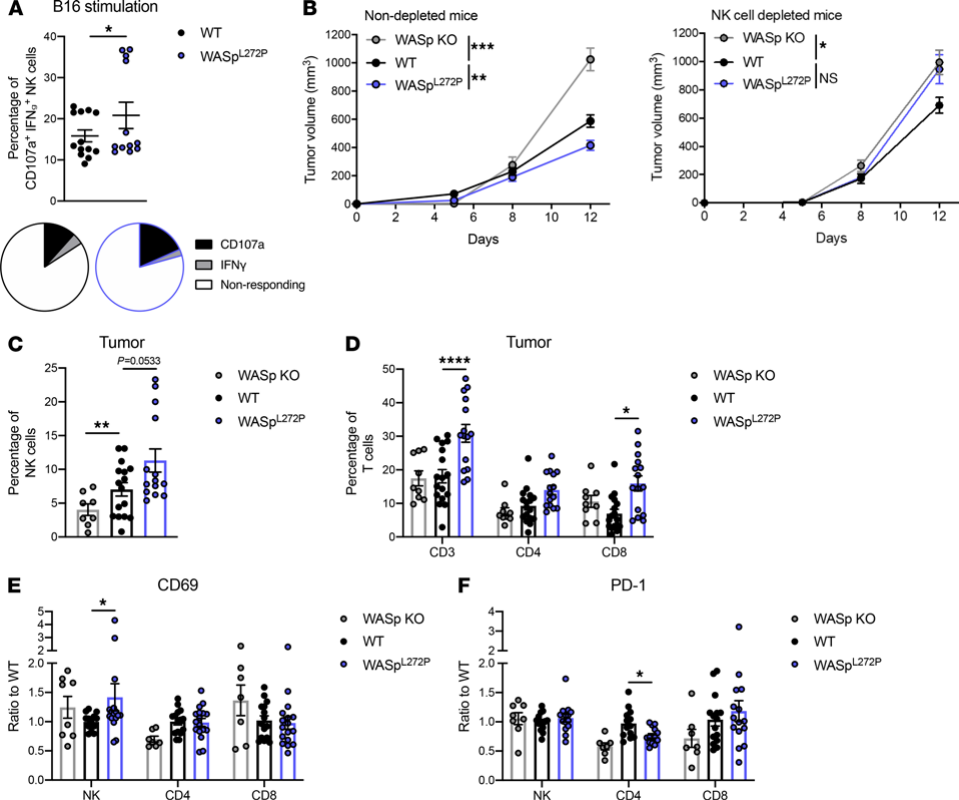
WASp^L272P^ mice exhibit increased responsiveness against tumors and have a higher infiltration of activated NK and T cells. (**A**) Degranulation and IFN-γ production of WT and WASp^L272P^ NK cells against B16 melanoma cells in vitro. CD107a^+^IFN-γ^+^ NK cells include CD107a SP, IFN-γ SP, and CD107a IFN-γ DP cells. The data represent 2 individual experiments combined. Each dot represents 1 mouse. WT *n* = 13, WASp^L272P^ *n* = 12. Graph shows mean values ± SEM and significance was assessed by 2-tailed Student’s *t* test and the Mann-Whitney correction. The average of nonresponding and CD107a/IFN-γ–responding NK cells after B16 coculture is also shown as pie charts in each genotype. (**B**) Tumor growth of subcutaneous B16 melanoma in nondepleted (*left*) and NK cell–depleted (*right*) WASp-KO, WT, and WASp^L272P^ mice. The data represent 5 individual experiments combined for the nondepleted mice and 2 for the depleted mice. Nondepleted: WASp-KO *n* = 10, WT *n* = 31, WASp^L272P^ *n* = 27; depleted: WASp-KO *n* = 12, WT *n* = 10, WASp^L272P^ *n* = 11. Graphs show mean values ± SEM and significance was assessed by 1-way ANOVA. (**C**) NK cell and (**D**) T cell infiltration into the tumor of WASp-KO, WT, and WASp^L272P^ mice, shown as percentage of alive, single lymphocytes. (**E**) Expression of activating receptor CD69 and (**F**) inhibitory receptor PD-1 on tumor-infiltrating NK and T cells in B16 tumors from WASp-KO, WT, and WASp^L272P^ mice. The data represent 3 individual experiments combined. Each dot represents 1 mouse. WASp-KO *n* = 8, WT *n* = 16, WASp^L272P^ *n* = 14. Graph shows mean values ± SEM and significance was assessed by 1-way ANOVA. *P ≤ 0.05, **P ≤ 0.01, ***P ≤ 0.001, ****P ≤ 0.0001. If no other indication, results were not significant.

To examine the tumor surveillance capacity in the mouse model of XLN, we used syngeneic B16 melanoma cells, which have low expression of MHC class I molecules (45). We first examined the NK cell response to B16 melanoma cells in vitro after a 4-hour coculture. When compared with WT NK cells, WASp^L272P^ NK cells showed higher degranulation, as measured by CD107a surface expression (Figure 7A). We next injected B16 melanoma cells subcutaneously and measured the tumor size during a period of 12 days. At day 12, the B16 melanoma cells formed larger tumors in WT mice whereas the tumors of WASp^L272P^ mice had smaller size (Figure 7B, solid lines). WASp-KO mice had an increased growth of B16 melanoma compared with WT, as shown previously (Figure 7B, solid lines, ref. 32). To specifically address the role of NK cells in the tumor progression of WASp^L272P^ mice, NK cells were depleted by intraperitoneal injection of anti-NK1.1 antibody. The absence of NK cells was confirmed by flow cytometry of peripheral blood (Supplemental Figure 8A). Devoid of an NK cell response, WASp^L272P^ mice displayed a tumor growth curve indistinguishable from WASp-KO mice (Figure 7B), underlining the importance of NK cells in the tumor immunosurveillance of WASp^L272P^ mice. WT mice that received NK cell depletion antibodies showed a lower tumor size, compared with WASp^L272P^ mice, suggesting an important role for CD8^+^ T cell responses in these mice (Figure 7B). We next examined the immune cell infiltrates of the tumors on day 12. Interestingly, the smaller B16 tumors of WASp^L272P^ mice contained a larger infiltration of NK cells, when compared with WT tumors (Figure 7C). WASp^L272P^ mice also had an increased percentage of tumor-infiltrating T cells, particularly CD8^+^ T cells (Figure 7D). The increased T cell infiltration observed in WASp^L272P^ mice was completely abrogated in WASp^L272P^ mice depleted of NK cells (Supplemental Figure 8B). Tumor-infiltrating NK cells in WASp^L272P^ mice had a higher expression of the activating marker CD69 (Figure 7E), suggesting increased activation of WASp^L272P^ NK cells. CD4^+^ T cells in WASp^L272P^ tumors had a decreased expression of inhibitory receptor PD-1 (Figure 7F). No difference in expression of the activating receptor NKG2D was observed (Supplemental Figure 8C). These data suggest that WASp^L272P^ NK and T cells showed an activated phenotype in the tumor microenvironment.

Taken together, our data suggest that NK cells of the XLN mouse model had an increased capacity to control aggressive tumor growth and to recognize and respond to loss of MHC class I molecules on target cells.

## Discussion

Studies of WASp-deficient NK cells and CTLs have revealed the important role for WASp-mediated actin dynamics in cytotoxic cells for eradication of tumor cells (15, 30–34). WAS patients are prone to developing malignancies of poor prognosis and most frequently lymphoreticular tumors including non-Hodgkin lymphoma (76% of the total tumors associated with WAS), Hodgkin lymphoma, and Burkitt lymphoma (46–50). Both reduced tumor immunosurveillance by cytotoxic cells (15, 30–34, 37–39) and intrinsic cell transformation (15, 51, 52) contribute to malignancies in WAS. In the family with WASp^L270P^ XLN (1), 2 out of 6 affected males have developed myelodysplastic syndrome and leukemia, and 1 unrelated patient with the WASp^I294T^ mutation developed myelodysplastic syndrome (2). In a large family with XLN with the WASp^I294T^ mutation, no malignant events have been reported so far (8). Genetic abnormalities have been detected in patient and murine XLN B cells and are associated with an increased load of polymerized actin leading to faulty cell division (10, 12, 16). Expression of WASp XLN mutations in cell lines leads to increased viscosity of the cells, suggesting that increased load of polymerized actin may be disadvantageous to cell functionality (16). In XLN neutrophils, we recently found that the increased polymerized actin is dynamic, leading to increased rearrangement of the actin cytoskeleton and increased adhesive and migratory capacity of the cells in vitro and in vivo (11). Our and other previous studies have revealed that cytotoxic cells in patients with WAS and WASp-KO mice are hyporesponsive (15, 30–33). We here examined how mutations that render WASp constitutively active in XLN patients, as opposed to loss-of-function mutations in WAS, affect cytotoxic cell activity and function. By examination of XLN patient and murine cells, our data show that XLN cytotoxic cells had normal to higher capacity to respond to and kill tumor cells. These data suggest that the increased susceptibility to malignancies in patients with XLN is not caused by dysfunctional responses by individual cytotoxic cell populations, but rather by NK cell cytopenia and increased susceptibility to genetic instability of hematopoietic cells expressing constitutively active WASp (10, 12, 16). It is still possible that other NK cell functions and memory responses, not examined here, are affected in XLN patients.

Here, we report a phenotypical and functional characterization of NK and T cells from XLN patients carrying the WASp^L270P^ mutation and an XLN mouse model. As previously described (1, 2, 8), WASp^L270P^ patients had reduced NK cell numbers in peripheral blood and a skewed CD8^+^/CD4^+^ T cell ratio. Interestingly, the XLN patients showed a near absent CD56^bright^ NK cell population, similar to the phenotype observed in GATA2-deficient patients (53). However, in contrast to patients devoid of GATA2 who display a reduced NK cell cytotoxic response (53), we show that XLN NK cells had an increased capacity to respond to stimulation by degranulation and cytokine production. The discrepancy between lower NK cell numbers in XLN patient blood and the increased NK cell responsiveness could be explained by 2 scenarios. First, one consequence of the observed hyperactive phenotype could be an increased migratory capacity, which would lead to the cells being present in tissues rather than in peripheral blood. Severe blood neutropenia is one of the major symptoms in XLN. However, we observed that neutrophils are present in normal numbers in patient saliva and at sites of inflammation in WASp^L272P^ mice (11), suggesting an increased migration of XLN neutrophils, and possibly other immune cells, into tissues. Our bone marrow chimera experiments showed that WASp^L272P^ NK cells and T cells had an increased reconstitution rate in the spleen. Moreover, WASp^L272P^ NK cells and T cells showed increased infiltration into the tumor mass. Together, these data suggest an increased migratory capacity of cytotoxic lymphocytes in XLN. Increased apoptosis has been observed in B and T cells from WASp^I296T^ XLN mice, which is associated with increased genomic instability (12). This could be due to the increased load of polymerized actin, resulting from the activating mutation, which renders cells more prone to apoptosis, potentially explaining the observed cytopenias in patients with XLN. Mature neutrophils are required for NK cell development (54); however, WASp^L272P^ mice do not present with spontaneous neutropenia (11). Moreover, we did not detect any changes in NK cell maturation and education in WASp^L272P^ mice (this study), contrary to the mice discussed in the paper by Jaeger et al. (54). Together, this suggests that the observed phenotype of NK cells and CD8^+^ T cells of WASp^L272P^ mice is cell intrinsic.

NK cells and CD8^+^ T cells from the patients, and even more strikingly CD4^+^ T cells, had a high intracellular granzyme B content. While this is reminiscent of an exhausted phenotype (55), we found that XLN patient T cells responded to PMA and ionomycin stimulation to a higher degree than T cells from healthy controls. The CD4^+^CD8^lo^ T cell population that emerged in the 2 XLN patients is potentially interesting. T cells expressing both CD4 and CD8 molecules have been described in several pathological conditions in humans, such as autoimmune disease, T and B cell lymphomas, leukemias, and infectious diseases (56, 57). This population is considered exceedingly cytotoxic and could represent a CD4^+^ T cell population with cytolytic activity, at least in some viral infections (57). This seems plausible in the XLN context as well, especially considering the high granzyme B content in the CD4^+^ T cells of the XLN patients and the fact that even though the patients are severely neutropenic, they do not have an overwhelming number of infections (1, 11).

To elucidate the role of NK and T cells with constitutively active WASp in immunosurveillance against tumors, we generated a mouse model of XLN harboring the corresponding WASp^L272P^ mutation. In vitro degranulation responses were increased in WASp^L272P^ NK cells. We reasoned that the elevated NK cell responses could be caused by an altered maturation and/or education of NK cells; however, WASp^L272P^ NK cells appeared phenotypically very similar to WT NK cells. The only exception was a decreased expression of the inhibitory receptor KLRG1 in WASp^L272P^ NK cells. This is interesting because WASp-deficient NK cells from mice and patients with WAS have increased expression of KLRG1 (33). KLRG1 binds to cadherins on target cells and is expressed on the most mature NK cells (58, 59). The role of KLRG1 has been associated with an exhausted phenotype; however, mature NK cells expressing KLRG1 are more efficient in the eradication of some tumor types (60). The implication for WASp in KLRG1 signaling remains to be determined. CD4^+^ and CD8^+^ T cells from WASp^L272P^ mice were phenotypically indistinguishable from WT T cells. Our previous studies identified that T cells have an increased quantity of polymerized actin because of constitutively active WASp (12). Murine XLN T cells migrate normally in vitro and have a normal proliferative response but a decreased spreading response (12).

The question remained whether increased actin polymerization would allow for the NK and T cells to perform their cytotoxic functions. We studied an important part of the killing response, the formation of the immune synapse. WASp^L272P^ NK and CD8^+^ T cells formed functional lytic synapses characterized by polarization of granules and F-actin accumulation to the interface with the target cells. WASp-dependent actin networks have been shown to promote the stability of T cell lytic synapses and potentiate mechanical forces required for lytic granule release (61, 62). When we examined the actin cytoskeleton at the lytic synapse in response to cross-linking anti-CD3/CD28 antibodies and quantitative imaging, WASp^L272P^ CD8^+^ T cells spread and accumulated more actin, indicating a more dynamic actin cytoskeleton. XLN patient T cells also displayed an interesting phenotype, with increased granzyme B content, degranulation, and cytokine production capacity. CD4^+^ and CD8^+^ T cells from WASp^L272P^ mice were able to degranulate, produce IFN-γ, and form conjugates to the same extent as WT T cells, suggesting a more moderate phenotype compared with WASp^L272P^ NK cells. Using specific receptor engagement on glass surfaces and quantitative imaging, WT and WASp^L272P^ NK cells showed similar spreading and F-actin accumulation in response to NKp46 activation, whereas WASp-KO NK cells had a reduced response. This was remarkable given the data from XLN patient NK cells showing increased granzyme B content and increased degranulation and IFN-γ production in response to PMA and ionomycin as well as enhanced cytotoxicity in vivo. Interestingly, WT and WASp-KO NK cells showed increased adhesion characterized by spreading and F-actin accumulation in response to LFA-1 engagement, while WASp^L272P^ NK cells failed to respond to this stimulation. WASp is required for actin accumulation at the site of LFA-1 ligation in NK cells (63). Signaling through LFA-1/ICAM-1 is sufficient to promote lytic granule convergence (43), thereby decreasing the potential of bystander killing due to nontargeted granule release (64). The lower response to ICAM-1 stimulation by WASp^L272P^ NK cells indicates that controlled regulation of WASp activation is important for steering convergent granule release. Moreover, LFA-1 signaling is used by T cells to lower activation thresholds (65), and WASp-dependent actin networks were also shown to contribute to nanocluster organization of LFA-1 in T cells (37). WASp-KO T cells had a variable response to ICAM-1 stimulation, as previously reported (37). Taken together, our data show that constitutively active WASp regulates actin dynamics downstream of bona fide activating receptors as well as LFA-1 and that cell type–specific usage of actin polymerization pathways leads to differential effects for functionality of NK and T cells.

Importantly, when assessing in vivo tumor responses, WASp^L272P^ mice had increased capacity to control growth of the aggressive B16 melanoma tumor when compared with WT mice. The tumor mass of WASp^L272P^ mice contained a substantially higher proportion of NK and T cells, and they displayed an activated phenotype. As previously reported, WASp-KO mice had increased tumor growth (32) with lower infiltration of NK cells. The absence of NK cells in WASp-KO mice did not affect tumor size, indicating the severe dysfunction of these cells in WASp deficiency (33). On the contrary, control of B16 tumors was highly dependent on NK cells in WASp^L272P^ mice whereas in WT mice, NK cell depletion had a smaller effect on tumor growth, suggesting involvement of other immune cells, such as CD8^+^ T cells, in the WT setting. Interestingly, NK cell depletion abrogated the increased T cell infiltration into tumors of WASp^L272P^ mice, suggesting a potential crosstalk between NK and T cells in the context of constitutively active WASp. Our data on immune synapse formation and how NK and T cells respond differently to stimuli by polarizing their actin cytoskeleton could further explain this WASp dependency. When compared with WT mice, WASp^L272P^ NK cells showed a faster rejection of MHC class I–deficient splenocytes. Together, these data lead us to propose that NK and T cells expressing constitutively active WASp in patients and in a mouse model of XLN display altered WASp-dependent actin dynamics and response to tumors. It is interesting that the WASp^L272P^ mice show a normal tumor rejection response to YAC-1 lymphoma cells. We believe that this stems from the role of IL-2 in WASp-independent activation of cytotoxic responses of NK cells (31, 33, 34). WASp-KO mice, despite having reduced cytotoxicity of NK cells and poor rejection capacity of MHC class I–negative cells and B16 melanoma cells, can control growth of lymphoma cells (YAC-1, A20, and RMA-S) in vivo (33). We reason that this could be due to tumor cells of lymphoid origin expressing multiple activating ligands and/or high quantity of cytokines, such as IL-2, that rescue the dysfunctional phenotype of WASp-KO NK cells. In contrast, B16 melanoma cells are not of hematopoietic cell origin and lack expression of IL-2 (33). We consider that the B16 melanoma cells and MHC class I–negative cells are more sensitive models to examine the effect of WASp activity in tumor cell eradication. We conclude that the symptoms observed in patients with XLN are likely not due to the inability of NK and T cells to respond to stimuli, such as the emergence of tumors. On the contrary, our data reveal that increased WASp activity may be advantageous for cytotoxic cell functionality.

## Methods

Further information can be found in Supplemental Methods.

### Patients.

Peripheral blood cells were analyzed from 2 sibling XLN patients (1), 1 carrier mother, 1 sister with unknown carrier status, and 2 healthy individuals who were sex- and age-matched to the XLN patients, serving as the control group. The mother and sister were grouped together and referred to as mother/sister. The blood was anticoagulated by potassium EDTA or sodium heparin. PBMCs were isolated using the Ficoll isolation method, and NK and T cells were examined by flow cytometry. Blood from all participants was collected at 2 distinct time points and data represent both experiments combined.

### Mice.

WASp^L272P^ mice on the C57BL/6 (H-2^b^) background were recently described (11). All animals used, WASp-KO, WASp^L272P^, WT littermate control, LifeActGFP, and β_2_ microglobulin–KO (β2m^−/−^; H-2^b^ deficient) mice, were bred and maintained at the animal facility of the Department of Microbiology, Tumor and Cell Biology and the KM-Wallenberg facility at Karolinska Institutet under specific pathogen–free conditions. Mice were used at 6 to 12 weeks of age.

### Bone marrow chimeras.

For generation of mixed bone marrow chimeras, 1 × 10^7^ WASp^L272P^ or WASp-KO bone marrow cells (expressing CD45.2) were mixed with WT bone marrow cells (expressing CD45.1) at a 3:1 ratio and transplanted by intravenous injection into irradiated (13 Gy) WT C57BL/6 recipient animals. The ratio 3:1 is based on the strong disadvantage in the lymphocyte lineage of WASp-KO cells when compared with WT cells (11, 66). Spleens were harvested for flow cytometry analysis 8–10 weeks after bone marrow reconstitution. The reconstitution ratio was calculated as (mutant cells in sample/WT cells in sample)/(mutant cells in inoculate/WT cells in inoculate), transformed, and plotted on a log_2_ scale. Relative reconstitution ratio log_2_[(Mut/WT)/(Mut/WT)_graft_] is shown on the *y* axis of Figure 6C.

### In vivo tumor growth.

B16 melanoma cells (1 × 10^5^) were injected into the right flank of WT and WASp^L272P^ C57BL/6 mice in Matrigel (BD Biosciences) at a 1:1 cell solution/gel ratio. Mice were monitored for 12 days, and tumor size was measured with a caliper on days 5, 8, and 12. On day 12, tumors were isolated from mice and mechanically disrupted, and lymphocytes were separated from tumor cells by Ficoll separation. Tumor cells and tumor-infiltrating lymphocytes were analyzed by flow cytometry.

YAC-1 cells were used for in vivo imaging by labeling them with the lipophilic dye DiR (Thermo Fisher Scientific) according to the manufacturer’s instructions. YAC-1 cells (1 × 10^6^) were injected subcutaneously into WT and WASp^L272P^ mice. In vivo imaging was performed 3 hours, 9 hours, 24 hours, and 48 hours after injection. In vivo imaging of luminescence was performed with a charge-coupled device camera, mounted in a light-tight chamber (IVIS Spectrum CT, Caliper LifeSciences, PerkinElmer). Anesthesia was induced by 4% isoflurane and maintained by 2.3% isoflurane throughout the imaging procedure. Regions of interest were localized around the injection sites, and intensity was quantified as radiant efficiency in (p/s/cm^2^/sr)/(mW/cm^2^) and normalized at every time point by the initial fluorescence measurement. Analysis was performed using the Living Image software version 4.1 (Caliper Life Sciences, PerkinElmer).

### NK cell depletion.

For in vivo depletion of NK cells in C57BL/6 mice prior to B16 tumor development, 100 μg anti-NK1.1 antibody (clone PK136, Bio X Cell) was injected i.p. on days –3, +1, and +6, relative to tumor cell injection (day 0). NK cell depletion was confirmed by staining peripheral blood cells with anti-NKp46 and the absence of NKp46^+^CD3^–^ cells on day +7 (Supplemental Figure 8A).

### In vivo β2m^–/–^ rejection assay.

Splenic single-cell suspensions from WT and β2m^–/–^ C57BL/6 mice were labeled with a low (0.5 μM) and a high (5 μM) dose of CFSE (33, 67, 68). The cells were then mixed in a 1:1 ratio and injected intravenously at a total of 30 × 10^6^ cells into WT and WASp^L272P^ C57BL/6 mice. Blood was taken after 8 hours and 24 hours, and spleens were collected after 24 hours or 48 hours. The amount of rejected β2m^–/–^ cells was assessed by flow cytometry and calculated as: 100% – (β2m^–/–^ cells in sample/WT cells in sample)/(β2m^–/–^ cells in inoculate/WT cells in inoculate)%.

### Statistics.

All statistical analyses were performed using Prism software (versions 6–8, GraphPad). Results are expressed as mean ± SEM. All data were analyzed by ROUT (Q = 1.0%) for the exclusion of outliers. The statistical test used for each specific experiment is denoted in the figure legends. Generally, differences between 2 individual groups were analyzed for statistical significance using the nonparametric 2-tailed Student’s *t* test and Mann-Whitney correction, and differences between more than 2 groups were analyzed for statistical significance using the 1-way ANOVA test. NS, not significant (*P* > 0.05), *P ≤ 0.05, **P ≤ 0.01, ***P ≤ 0.001, ****P ≤ 0.0001.

### Study approval.

The Code of Ethics of the World Medical Association (Declaration of Helsinki) for human samples was followed, the study was approved by the Institutional Review Board of UZ Leuven (B322201629893), and a written informed consent form was obtained from all individuals. All animal experiments were performed according to the EU Directive 2010/63/EU for animal experiments and after approval from the local ethical committee (the north Stockholm district court, permits N77/13, N272/14, 11156-2018) and in accordance with national and institutional guidelines.

## Author contributions

JSK and LSW designed the research; JSK, MMSO, JR, MBS, SMN, MH, MK, AKW, SR, HB, and AS performed the experiments and analyzed the data; SKS, DPL, SBS, and KK contributed with critical tools; PV provided XLN patient samples and analyzed patient data; JSO supervised the imaging analysis; JSK and LSW wrote the manuscript; and all authors edited the manuscript.

## Supplementary Material

Supplemental data

## Version 1 02/23/2021

In-Press Preview

## Version 2 03/22/2021

Electronic publication
