# Supplementary material for: Constitutive activation of WASp leads to abnormal cytotoxic cells with increased granzyme B and degranulation response to target cells
Source: JCI Insight. 2021 Mar 22;6(6):e140273. doi: 10.1172/jci.insight.140273 (PMC8026198; doi:10.1172/jci.insight.140273)
Supplement: Supplemental data [file jciinsight-6-140273-s015.pdf]

Supplementary Methods and Figures

Constitutive WASp activation leads to abnormal cytotoxic cells with increased response against tumors

Joanna S. Kritikou[1], Mariana M.S. Oliveira[1], Julien Record[1], Mezida B. Saeed[1], Saket M. Nigam[1], Minghui He[1], Marton Keszei[1], Arnika K. Wagner[1], Hanna Brauner[1,2], Anton Sendel[1], Saikiran K. Sedimbi[1], Stamatina Rentouli[1], David P. Lane[1], Scott B. Snapper[3], Klas Kärre[1], Peter Vandenberghe[4], Jordan S. Orange[5], and Lisa S. Westerberg[1]

[1]Department of Microbiology Tumor and Cell Biology, Biomedicum C7, Karolinska Institutet, Stockholm 17177, Sweden; [2]Department of Medicine Solna, Division of Rheumatology, Center for Molecular Medicine, Karolinska Institutet, Stockholm 17177, Sweden; [3]Gastroenterology Division, Children's Hospital, Harvard Medical School, Boston MA 02115, USA; [4]Center for Human Genetics, University Hospital Leuven, Belgium, [5]Department of Pediatrics, New York Presbyterian Morgan Stanley Children's Hospital, Columbia University, Vagelos College of Physicians and Surgeons, New York 10032, USA.

Extended Methods

Antibodies and Flow Cytometry

For flow cytometry analysis of human samples, single-cell suspensions were labeled with fluorescently conjugated anti-human antibodies (clone shown in parenthesis): CD56 (HCD56), CD3 (OKT3), IFNγ (4S.B3), KLRG1 (2F1/KLRG1), CD69 (FN50), DNAM-1 (11A8) (all Biolegend), CD107a (eBioH4A3) (eBioscience), and Granzyme B (GB11) (Invitrogen). For flow cytometry analysis of murine cells, single-cell suspensions were labeled with fluorescently conjugated anti-mouse antibodies (clone shown in parenthesis): CD3 (145.2C11), CD27 (29A1.4), CD11b (M1/70.15), CD69 (H1.2F3), Ly6D (49-H4), CD244 (m2B4(B6)458.1), CD127 (A7R34), CD122 (TM-β1), Ly49A (YEI/48), DNAM-1 (TX42.1), CTLA-4 (UC10-4B9), LAG3 (C9B7W), (all Biolegend), NK1.1 (PK136), KLRG1 (2F1), CD107a (1D4B), IFNγ (XMG1.2), Ly49G2 (4D11), and NKG2D (CX5) (all BD Biosciences), NKp46 (29A1.4) (Life Technologies) and Ly49I (YLI-90), NKG2A (20d5) CD45.1 (A20), and CD45.2 (104) (eBioscience). The 4LO3311 hybridoma (for detection of Ly49C) was a kind gift from Suzanne Lemieux.

All surface staining was performed in phosphate-buffered saline (PBS) after blocking unspecific staining via FcγRII/III with purified anti-CD16/32 (clone 2.4G2 for mouse and clone 93 for human samples). Dead cells were excluded from gating with the Aqua dead cell dye (Life Technologies). For intracellular staining, cells were fixed and permeabilized with the Cytofix/Cytoperm kit (BD Biosciences) and then stained with antibodies for intracellular proteins. The gating strategy used for human and murine samples throughout the paper is shown in Supplementary Figure 1A. Murine NK cells are defined as $CD3^-NK1.1^+$ and T cells as $CD3^+NK1.1^-CD4^+$ or $CD3^+NK1.1^-CD8^+$, for CD4 and CD8 T cells respectively (Supplementary Figure 1B) Human NK cells are defined as $CD56^+CD3^-$ and subdivided into

$CD56^{bright}$ and $CD56^{dim}$ (Figure 1A) and T cells as $CD3^+CD56^-CD4^+$ or $CD3^+CD56^-CD8^+$, for CD4 and CD8 T cells respectively (Figure 1B). To better be able to compare results between experiments, the mean fluorescence intensity (MFI) was normalized to the average MFI of the WT samples in each experiment to generate an MFI to WT ratio shown in the Figures where indicated. All flow cytometry analyses were performed on an LSRFortessa X-20 (BD Biosciences) and data analysis was performed using the FlowJo software (Tree Star).

Cell culture and cell lines

Cell culture was performed at 37°C in a humidified atmosphere containing 5% CO2. Media was supplemented with 10% fetal calf serum (FCS), 100 U/ml penicillin, 100 µg/ml streptomycin, 0.2 mg/ml glutamine, and 50 µM 2-mercaptoethanol (referred to as complete media). All cell culture and stimulation of human and murine lymphocytes was performed in complete RPMI. B16-F10 melanoma cells (hereafter referred to as B16) was a kind gift from Thomas Tedder (Duke University) and YAC-1 lymphoma cells YAC-1 (ATCC® TIB-160™) were cultured in complete DMEM and RPMI, respectively.

In vitro NK and T cell stimulation

For stimulation of human samples, single-cell suspensions of PBMCs were incubated with the prototypical human NK cell target cell line K-562 (ATCC® CCL-243) to assess NK cell responses and with 50ng/ml PMA (Sigma) and 500ng/ml Ionomycin (Sigma) to assess NK cell and T cell responses. Media alone was used for unstimulated controls. Cells were incubated in complete RPMI medium for 6h, supplemented with 10µg/ml Brefeldin A (GolgiPlug; BD Biosciences) and anti-CD107a. Cells were then harvested and stained for surface markers and intracellular IFNγ and analyzed by flow cytometry. Degranulation was assessed by the surface expression of CD107a.

For stimulation of murine samples, WT and $WASp^{L272P}$ C57Bl/6 spleens were mechanically disrupted and erythrocytes were depleted with ACK buffer. For NK cell stimulations, cells were added to a plate pre-coated with 20μg/ml purified anti-NKp46 (R&D Systems) or 50μg/ml purified anti-NK1.1 (BD Biosciences) for 4h at 37°C. Media alone was used for unstimulated controls and PMA/Ionomycin for positive controls. The incubation was done in complete RPMI medium, supplemented with 10μg/ml Brefeldin A and anti-CD107a. For T cell stimulations, the cells were added to a plate pre-coated with 10μg/ml purified anti-CD3 (BD Biosciences) and 2μg/ml soluble purified anti-CD28 (Biolegend). Media alone was used for unstimulated controls and PMA/Ionomycin for positive controls. The incubation was done in complete RPMI medium for 4h or 3 days, and for the last 4h of the incubation 10μg/ml Brefeldin A and anti-CD107a were added. NK and T cells from WASp KO mice were used as negative controls since they do not respond to stimulation with antibodies against NK1.1 and NKp46 or CD3 and CD28, respectively. After the stimulations, cells were harvested, stained for surface markers and intracellular IFNγ and analyzed by flow cytometry. Degranulation was assessed by the surface expression of CD107a.

NK and T cell synapse formation and Imaging Flow Cytometry

The LifeActGFP mice that have GPF-tagged actin were crossed with WASp KO, WT, and $WASp^{L272P}$ and were used to assess synapse formation without the use of the phalloidin dye. For NK cell synapse formation, splenic NK cells from WASp KO, WT, and $WASp^{L272P}$ LifeActGFP C57Bl/6 mice were purified by negative selection MACS sorting (Miltenyi Biotech). NK cells were incubated with YAC-1 lymphoma cells at a 5:1 effector:target ratio for 1h at 37°C. YAC-1 lymphoma cells were pre-labelled with the SNARF-1 dye and NK cells with anti-NK1.1. Anti-CD3 and the Aqua dead cell dye (Life Technologies) were used to exclude contaminating T cells and dead cells. YAC-1/NK cell conjugates were fixed and permeabilized

(Cytofix/Cytoperm kit; BD Biosciences) and stained with anti-Granzyme B (GB12, Invitrogen). NK cell conjugates were gated according to their area and aspect ratio and as $CD3^{-}$ $NK1.1^{+}SNARF\text{-}1^{+}$ doublets. For T cell synapse formation, splenic CD4 and CD8 T cells from WASp KO, WT, and $WASp^{L272P}$ LifeActGFP C57Bl/6 mice were purified by negative selection MACS sorting (Miltenyi Biotech). Cells were pre-labelled with anti-CD4 and anti-CD8 and the Aqua dead cell dye and were then incubated with Dynabeads coated with anti-CD3 and anti-CD28 (Life Technologies), for 15 min at 37°C at a 1:1 T cell:bead ratio and then fixed (Cytofix/Cytoperm kit; BD Biosciences). T cells that formed conjugates with beads were gated as CD4 or CD8 and SSC^{high} since the beads have a high SSC intensity. Images were acquired on an $ImageStream^{X}$ Mark II (Amnis) and analysis was performed using the IDEAS v.6 software (Amnis).

Confocal, Stimulated Emission Depletion (STED) and Interference Reflection Microscopy (IRM)

Splenic NK, CD4 and CD8 T cells from WASp KO, WT, and $WASp^{L272P}$ C57Bl/6 mice were purified by negative selection using MACS sorting (Miltenyi Biotech). For the short-term imaging experiments cells were incubated for 10 min or 60 min in µ-Slide 8 Well Glass Bottom chambers (Ibidi, Cat.#:80827-90) coated with anti-NKp46 (R&D systems, Cat. AF2225), anti-CD3 and anti-CD28 (Biolegend, Cat. #: 317325 and 302933), or ICAM-1 (R&D systems, Cat. #796-IC) after being coated with 0.1mg/ml poly-L-lysine (PLL) (Invitrogen). The cells were then fixed, permeabilized, and labeled with phalloidin-Alexa Fluor 488 to detect F-actin. The imaging was performed on a Leica TCS SP5 microscope on reflection mode using the laser set at 633nm. The images were processed in FIJI/ImageJ: the original image was first duplicated, then the duplicated image was used to create a mask by successively using the functions enhanced contrast (5% saturation, normalised), gaussian blur (sigma:2 pixels) and auto-

threshold resulting in a binary image. The ROI of each cell was then extracted from the binary image using the analyse particles and added to the ROI manager. The ROIs were then used on the original image to measure the Area and F-actin integrated density (IntDen= area x mean gray value). For the long-term imaging experiments NK cells were incubated for 4h on glass coverslips coated with 20μg/mL anti-NKp46 (R&D Systems) after being coated with 0.1mg/ml poly-L-lysine (PLL) (Invitrogen). The cells were then fixed, permeabilized, and labeled with phalloidin-Alexa Fluor 488 to detect F-actin. The imaging was performed on a Leica TCS SP8 microscope with a time-gated Stimulated Emission Depletion (STED) and Interference Reflection Microscopy (IRM) module. Image analysis was performed on the Image J and CellProfiler software.

Live cell imaging

NK cells, CD4 and CD8 T cells were isolated from spleens of WT and WASp^{L272P} C57Bl/6 mice by negative magnetic selection using a cell-specific isolation kit (Miltenyi Biotec). YAC-1 lymphoma cells were used as NK cell targets (effector:target ratios 10:1 and 1:1) and A20 cells coated with anti-CD3 and/or anti-CD28 were used as T cell targets (effector:target ratio 10:1). Target cells were labelled with CFSE to distinguish from effector cells. Death was monitored by the appearance of cells positive for the Annexin V dye, present in the media during the co-culture on the IncuCyte S3 imaging system (Sartorius).

Supplementary Figure 1

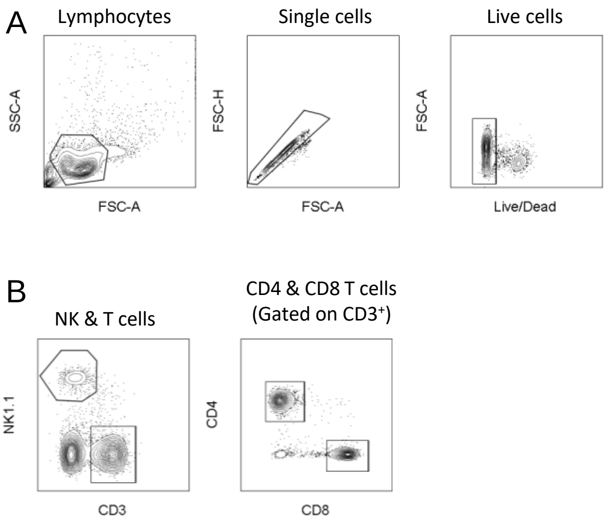

Supplementary Figure 1. Gating strategies. (A) Flow cytometry gating strategy for murine and human samples. **(B)** Flow cytometry gating strategy for murine NK cells and T cells (pre-gated on single, live lymphocytes).

Supplementary Figure 2

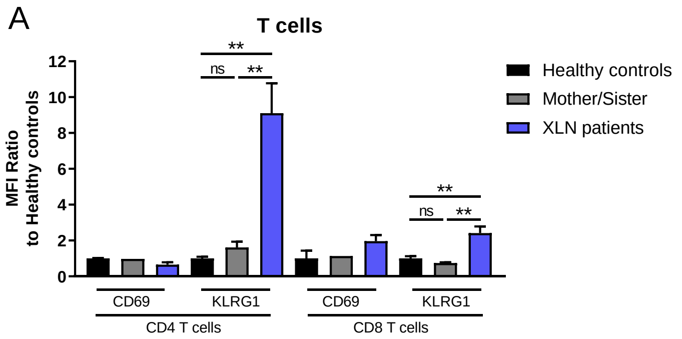

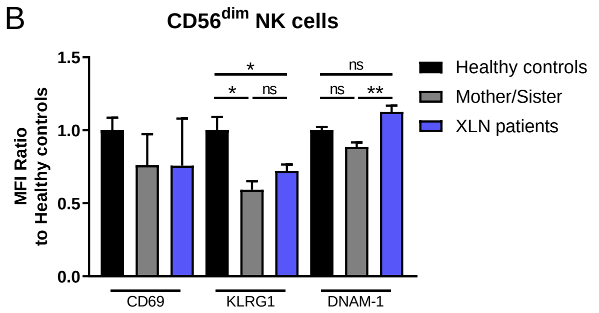

Supplementary Figure 2. Receptor expression in XLN patient NK and T cells. (A) Receptor expression of CD69 and KLRG1 on XLN patient CD4 and CD8 T cells. **B)** Expression of CD69, KLRG1 and DNAM-1 on XLN patient NK cells. The data represent 2 individual experiments combined. Graphs show mean values±SEM and significance was assessed by one-way ANOVA. * $p \leq 0.05$, ** $p \leq 0.01$. If no other indication, results were not significant.

Supplementary Figure 3

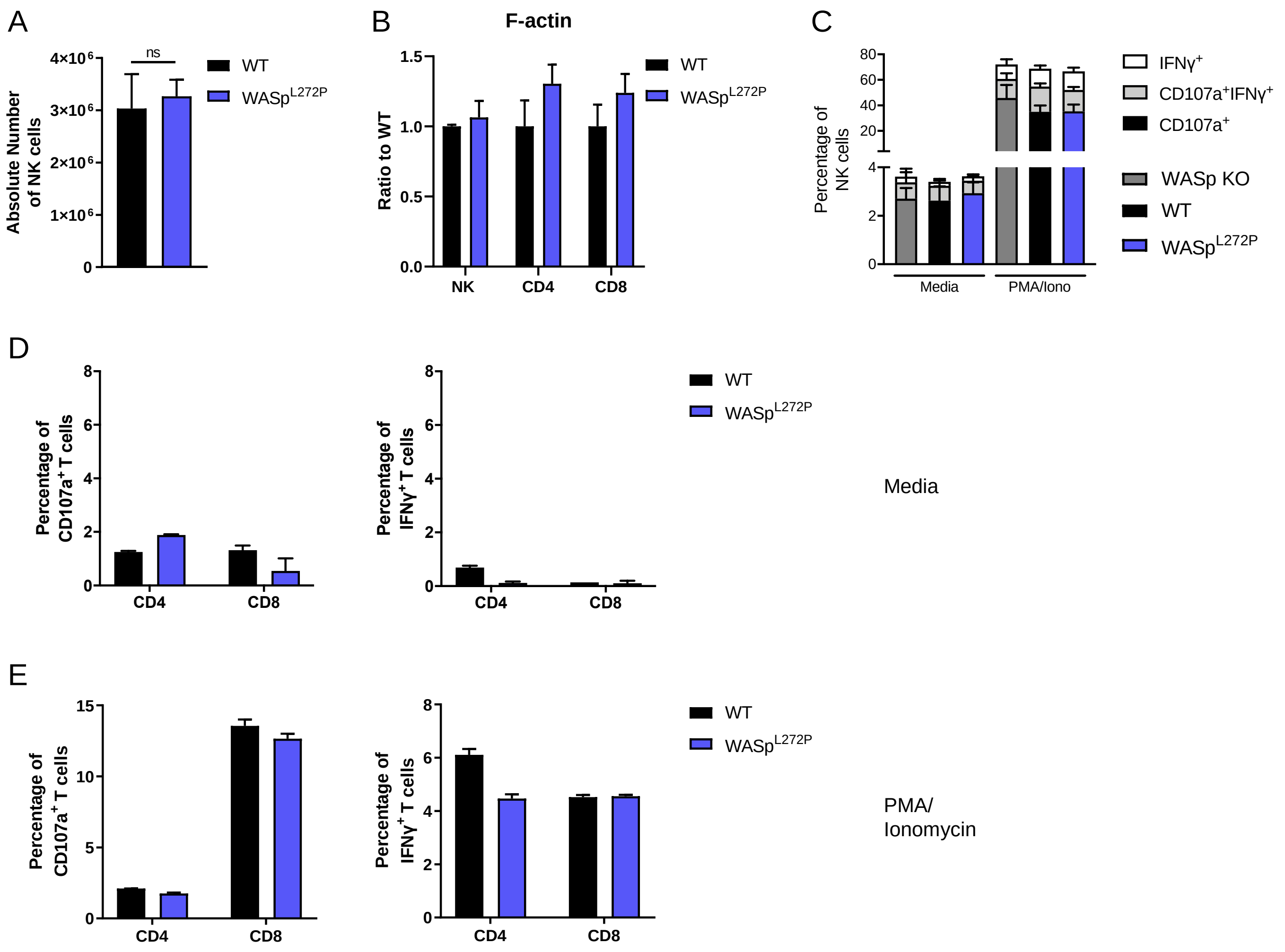

Supplementary Figure 3. Absolute cell number, F-actin content and control stimulations. **(A)** Absolute number of NK cells in spleens of WT and WASp^{L272P} mice. The analysis represents a pool of 3 individual experiments. WT n=13, WASp^{L272P} n=15. **(B)** F-actin content in NK cells, CD4 and CD8 T cells in WT and WASp^{L272P} mice as assessed by intracellular phalloidin staining and flow cytometry. The analysis represents a pool of 2 individual experiments. WT n=7, WASp^{L272P} n=8. **(C)** Degranulation and IFNγ production in NK cells from WASp KO, WT and WASp^{L272P} mice in media alone (negative control, left) and PMA/Ionomycin (positive control, right). A cumulative graph of 5 experiments is shown. WASp KO n=6, WT n=18, WASp^{L272P} n=17. **(D)** Degranulation and IFNγ production in CD4 and CD8 T cells from WT and WASp^{L272P} mice in media alone (negative control). The Y-axis scale corresponds to that of the anti-CD3/CD28 stimulation in Figure 4. **(E)** Degranulation and IFNγ production in CD4 and CD8 T cells from WT and WASp^{L272P} mice in PMA/Ionomycin positive control. One representative experiment is shown, n=4. Graphs show mean values±SEM and significance was assessed by Student's t test and the Mann-Whitney correction. ns: not significant. If no other indication, results were not significant.

Supplementary Figure 4

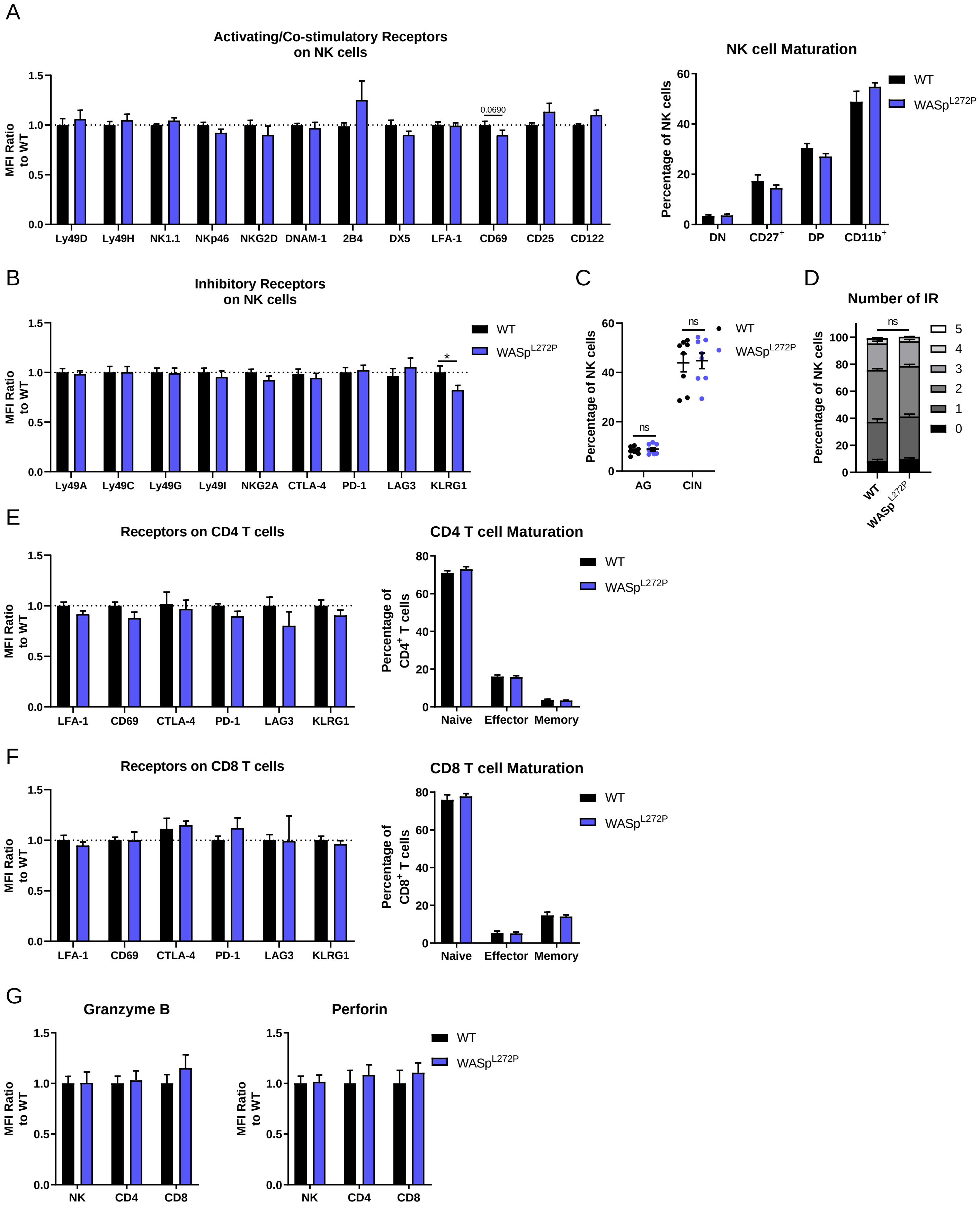

Supplementary Figure 4. Murine WASp^{L272P} NK cells and T cells have normal receptor expression and maturation status. (A-D) Analysis of the activating and inhibitory receptor repertoire in WASp^{L272P} NK cells. **(A)** Expression of activating/co-stimulatory receptors Ly49D, Ly49H, NK1.1, NKp46, NKG2D, DNAM-1, 2B4, DX5, LFA-1, CD69, CD25 and CD122 on WASp^{L272P} NK cells (*left*). Data from 3 individual experiments. WT n=10, WASp^{L272P} n=10. NK cell maturation assessed with markers CD11b and CD27 (*right*). The analysis is a pool of 4 individual experiments. WT n = 11, WASp^{L272P} n = 14. Graphs show mean values±SEM and significance was assessed by Student's t test and the Mann-Whitney correction. DN: CD11b^{-}CD27^{-}, DP: CD11b^{+}CD27^{+}. **(B-D)** Analysis of the inhibitory receptor repertoire to assess NK cell education in WASp^{L272P} C57Bl/6 mice. **(B)** Expression of inhibitory receptors Ly49A, Ly49C, Ly49G, Ly49I, NKG2A, CTLA-4, PD-1, LAG and KLRG1 on WASp^{L272P} NK cells. Data from 3 individual experiments. WT n=10, WASp^{L272P} n=10. **(C)** The number of NK cell inhibitory receptors reacting to self (CIN) or non-self (AG) MHC Class I in C57Bl/6 hosts. The two classes of inhibitory receptors; CIN = Ly49C, Ly49I and/or NKG2A, AG = Ly49A and/or Ly49G2. Each dot represents one mouse. WT n=8, WASp^{L272P} n=8. Graph shows mean values±SEM and significance was assessed by Student's t test and the Mann-Whitney correction. **(D)** The number of different inhibitory receptors per NK cell. WT n=8, WASp^{L272P} n=8. Graph shows mean values±SEM and significance was assessed by Student's t test and the Mann-Whitney correction. **(E-F)** Analysis of the activating and inhibitory receptor repertoire in WASp^{L272P} T cells. **(E)** Expression of receptors LFA-1, CD69, CTLA-4, PD-1, LAG3 and KLRG1 on CD4 T cells in WASp^{L272P} mice (*left*). Data from 3 individual experiments. WT n=10, WASp^{L272P} n=10. CD4 T cell maturation assessed by markers CD44 and CD62L (*right*). Naïve: CD44^{low}CD62L^{hi}, Effector: CD44^{low}CD62L^{low}, Memory: CD44^{hi}CD62L^{low}. Data from 4 individual experiments. WT n=11, WASp^{L272P} n=14. Graphs show mean values±SEM and significance was assessed by Student's t test and the Mann-Whitney correction. **(F)** Expression of receptors LFA-1, CD69, CTLA-4, PD-1, LAG3 and KLRG1 on CD8 T cells in WASp^{L272P} mice (*left*). Data from 3 individual experiments. WT n=10, WASp^{L272P} n=10. CD4 T cell maturation assessed by markers CD44 and CD62L (*right*). Naïve: CD44^{low}CD62L^{hi}, Effector: CD44^{low}CD62L^{low}, Memory: CD44^{hi}CD62L^{low}. Data from 4 individual experiments. WT n=11, WASp^{L272P} n=14. Graphs show mean values±SEM and significance was assessed by Student's t test and the Mann-Whitney correction. (**G**) Expression of Granzyme B and perforin in NK cells, CD4 T cells, and CD8 T cells in WT and WASp^{L272P} mice (*left*). Data from 3 individual experiments. WT n=10, WASp^{L272P} n=10. Graphs show mean values±SEM and significance was assessed by Student's t test and the Mann-Whitney correction. * $p \leq 0.05$, and ns: not significant. If no other indication, results were not significant.

Supplementary Figure 5

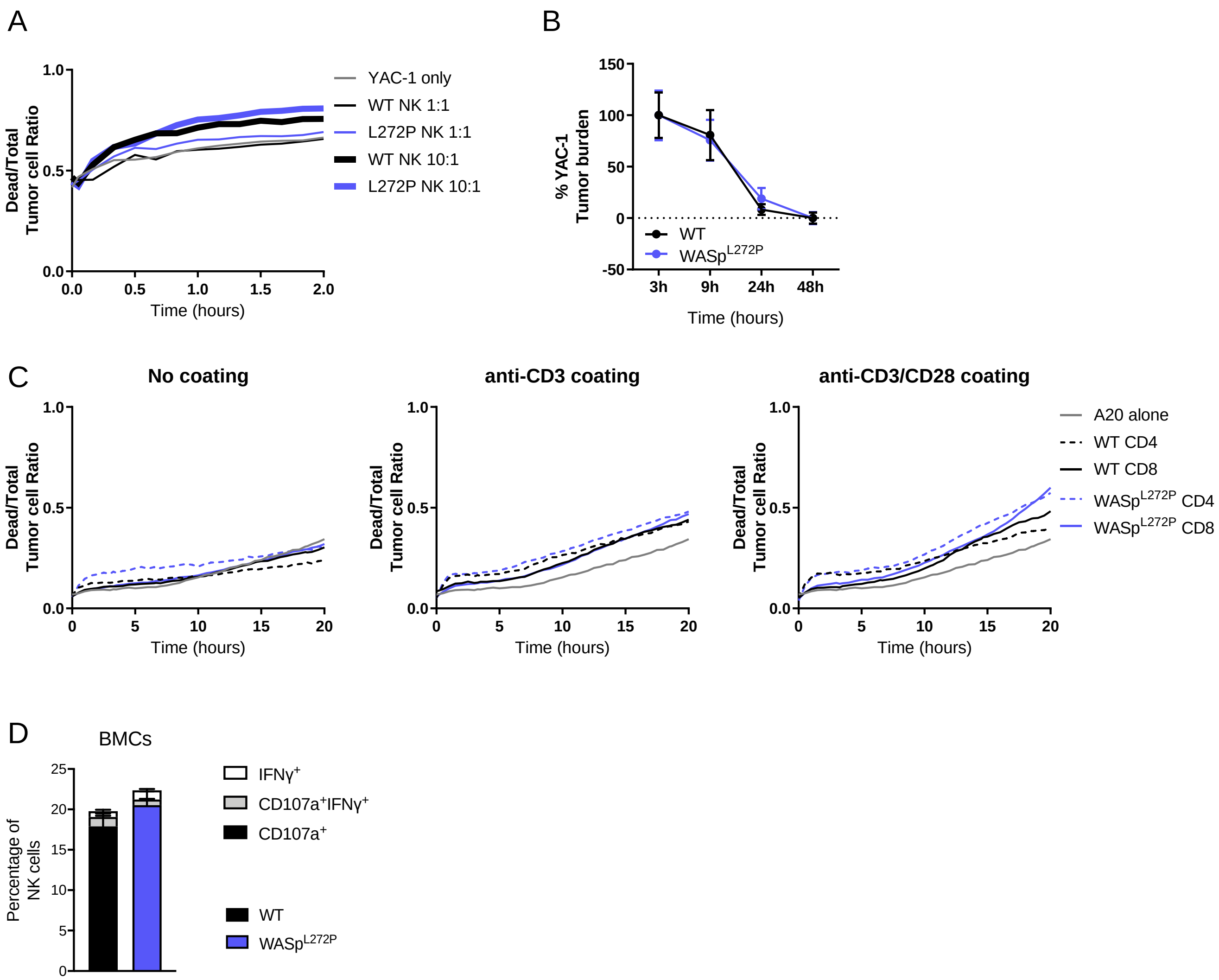

Supplementary Figure 5. WASp^{L272P} NK cell and T cell responses against tumor cells. (A) *In vitro* live cell imaging of WT and WASp^{L272P} NK cell-mediated killing of YAC-1 lymphoma cells. A representative experiment of 3 is shown. (**B**) *In vivo* imaging of of WT and WASp^{L272P} C57Bl/6 mice, subcutaneously injected with 1×10^6 YAC-1 lymphoma cells that were labelled with the DiR dye to assess NK-mediated tumor cell rejection. YAC-1 lymphoma cell rejection is shown as a function of percentage of remaining YAC-1 fluorescence in WT and WASp^{L272P} mice at each time point. Time points shown are 3h, 9h, 24h and 48h after injection, from a pool of 5 individual experiments. WT n=17, WASp^{L272P} n=18. **(C)** Live cell imaging of WT and WASp^{L272P} CD4 and CD8 T cell-mediated killing of A20 target cells. Target cells have either not been coated (*left*), coated with anti-CD3 (*middle*), or coated with anti-CD3 and anti-CD28 (*right*). A representative experiment of 3 is shown. Graph shows mean values±SEM and significance was assessed by one-way ANOVA. If no other indication, results were not significant. **(D)** The stimulation data of the bone marrow chimaeras (BMC) from Figure 6B shown as bar graphs.

Supplementary Figure 6

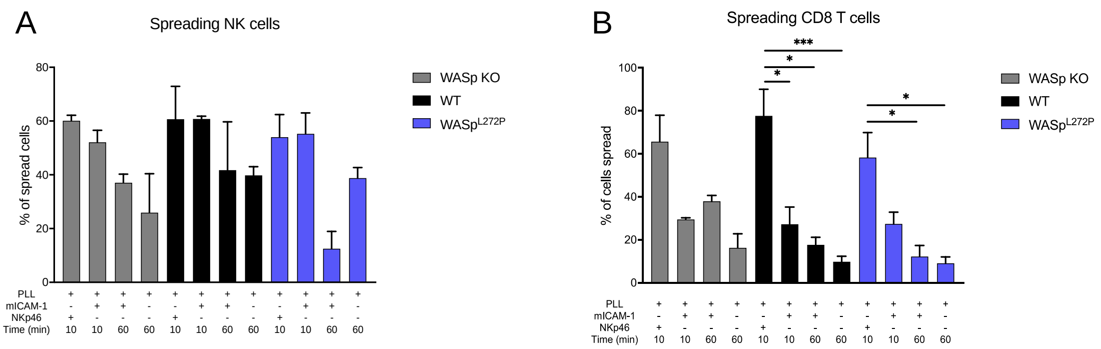

Supplementary Figure 6. Adhesion of NK and CD8 T cells to ligand coated coverslips. Quantification of the proportion of cells adhering to Poly-L-Lysine (PLL), crosslinking antibodies or recombinant ligands following incubation of (A) NK cells and (B) CD8 T cells for indicated times and used for quantitative analysis of F-actin polarization in Figure 5. Adhesion in this assay was scored from Interference Reflection Microscopy (IRM) images. Two individual experiments combined are shown. For each condition, between 200 and 400 cells were analyzed. Graphs show mean values±SEM and significance was assessed by one-way ANOVA. * $p \leq 0.05$, *** $p \leq 0.001$, **** $p \leq 0.0001$ and ns: not significant.

Supplementary Figure 7

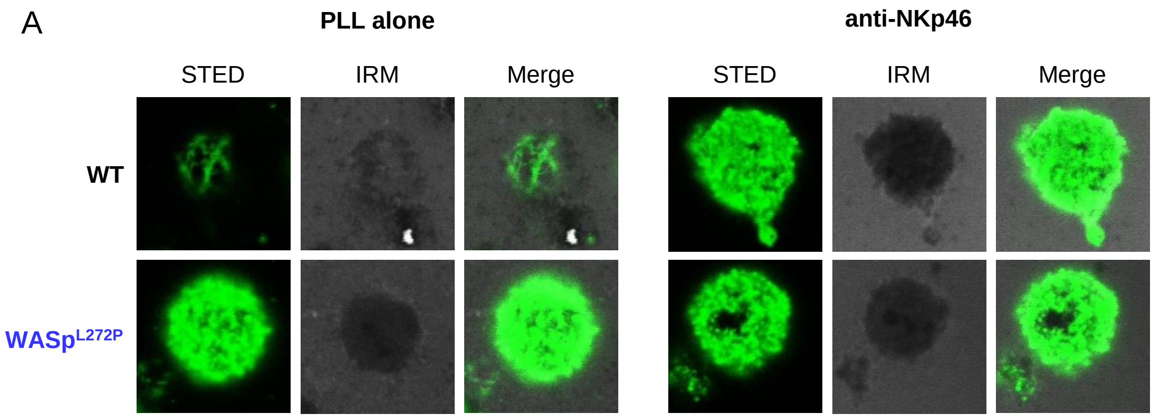

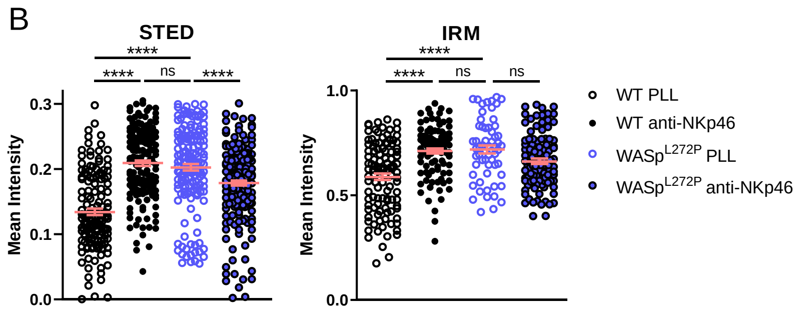

Supplementary Figure 7. F-actin density measured by STED imaging and surface attachment by IRM microscopy of WT and WASp^{L272P} NK cells after 4h. (A) Representative STED images of the NK cell F-actin accumulation towards a surface coated with PBS (*left*) or anti-NKp46 (*right*) and IRM images. **D)** Graphs showing quantification of F-actin MFI at the PBS or anti-NKp46-coated surface (STED, *left*) and quantification of the intensity of attachment (IRM, *right*), both in arbitrary units. The data represent 3 individual experiments combined. Dots indicate pictures analyzed, WT PBS n=287, WT NKp46 n=214, WASp^{L272P} PBS n=90, WASp^{L272P} NKp46 n=63, from a total of WT n=9, WASp^{L272P} n=6, and WASp^{I296T} n=5 mice. Graphs show mean values±SEM and significance was assessed by Student's t test and the Mann-Whitney correction. Graphs show mean values±SEM and significance was assessed by one-way ANOVA. * $p \leq 0.05$, *** $p \leq 0.001$, **** $p \leq 0.0001$ and ns: not significant. Abbreviations: STED; Stimulated Emission Depletion, IRM; Interference Reflection Microscopy.

Supplementary Figure 8

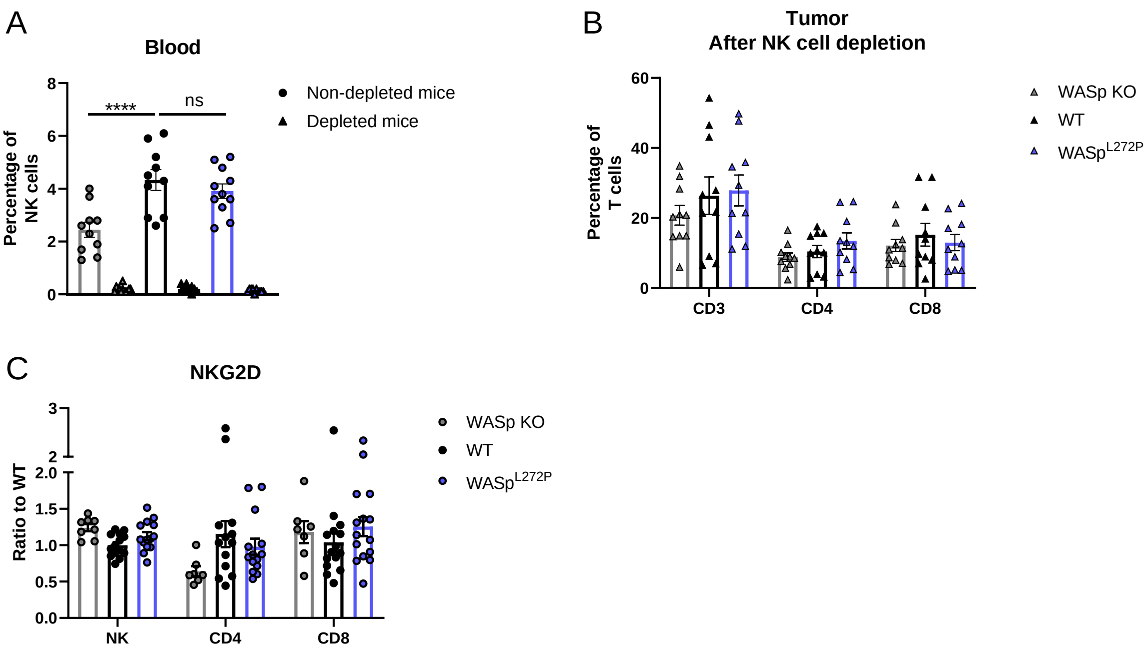

Supplementary Figure 8. NK cell depletion verification and NKG2D expression on tumor-infiltrating cells. (A) NK cell percentage in the blood of WASp KO, WT and WASp^{L272P} B16 tumor-bearing mice injected with or without depleting antibodies against NK1.1. **(B)** T cell percentages in B16 tumors of WASp KO, WT and WASp^{L272P} mice depleted of NK cells. The data represent 2 individual experiments combined. Each dot represents one mouse. Non-depleted: WASp KO n=10, WT n=31, WASp^{L272P} n=27, depleted: WASp KO n=10, WT n=10, WASp^{L272P} n=10. **(C)** NKG2D expression on tumor infiltrating NK cells and T cells in B16 tumors from WASp KO, WT and WASp^{L272P} mice. The data represent 3 individual experiments combined. Each dot represents one mouse. WASp KO n=8, WT n=16, WASp^{L272P} n=14. Graphs show mean values±SEM and significance was assessed by one-way ANOVA. **** $p \leq 0.0001$ and ns: not significant. If no other indication, results were not significant.
